# Gli1 Mediates Lung Cancer Cell Proliferation and Sonic Hedgehog-Dependent Mesenchymal Cell Activation

**DOI:** 10.1371/journal.pone.0063226

**Published:** 2013-05-07

**Authors:** Olga Bermudez, Elisabeth Hennen, Ina Koch, Michael Lindner, Oliver Eickelberg

**Affiliations:** 1 Comprehensive Pneumology Center, University Hospital of the Ludwig-Maximilians-University Munich and Helmholtz Zentrum München, Member of the German Center for Lung Research, Munich, Germany; 2 Center for Thoracic Surgery, Asklepios Biobank for Lung Diseases, Comprehensive Pneumology Center, Asklepios Clinic Munich-Gauting and Ludwig Maximilians University Munich, Gauting, Germany; National Cancer Center, Japan

## Abstract

Non-Small-Cell-Lung-Cancer (NSCLC) represents approximately 85% of all lung cancers and remains poorly understood. While signaling pathways operative during organ development, including Sonic Hedgehog (Shh) and associated Gli transcription factors (Gli1-3), have recently been found to be reactivated in NSCLC, their functional role remains unclear. Here, we hypothesized that Shh/Gli1-3 could mediate NSCLC autonomous proliferation and epithelial/stromal signaling in the tumoral tissue. In this context, we have investigated the activity of Shh/Gli1-3 signaling in NSCLC in both, cancer and stromal cells. We report here that inhibition of Shh signaling induces a significant decrease in the proliferation of NSCLC cells. This effect is mediated by Gli1 and Gli2, but not Gli3, through regulation of cyclin D1 and cyclin D2 expression. While exogenous Shh was unable to induce signaling in either A549 lung adenocarcinoma or H520 lung squamous carcinoma cells, both cells were found to secrete Shh ligand, which induced fibroblast proliferation, survival, migration, invasion, and collagen synthesis. Furthermore, Shh secreted by NSCLC mediates the production of proangiogenic and metastatic factors in lung fibroblasts. Our results thus provide evidence that Shh plays an important role in mediating epithelial/mesenchymal crosstalk in NSCLC. While autonomous Gli activity controls NSCLC proliferation, increased Shh expression by NSCLC is associated with fibroblast activation in tumor-associated stroma. Our study highlights the relevance of studying stromal-associated cells in the context of NSCLC regarding new prognosis and therapeutic options.

## Introduction

Lung cancer is the deadliest cancer worldwide. Currently, no effective treatment options exist for lung cancer and the 5-year survival rate is only 14% for patients with treatment [1].The lack of effective long-term therapy is related with the complexity of lung cancer and hence with the need for better understanding the biology of lung carcinogenesis. Little attention has thus far been addressed to the tumor-surrounding microenvironment, which constitutes the tumor-associated stroma and acts as active participant in tumorigenesis. In the past years, growing evidence has pointed out the importance of the stroma in tumor initiation, progression and metabolism of many types of cancer [2]–[7]. Likewise, signaling in the stromal cells has been shown to be essential for the malignant transformation of epithelial cells [2], [5], [6].

Pathways involved in organogenesis and lung branching morphogenesis, including Hedgehog (Hh) signaling, have been recently identified as key players in human cancers [8]. Three Hedgehog (Hh) genes exist in mammals, with Sonic Hedgehog (Shh) as the most broadly expressed gene. Secreted Shh binds to the receptors Patched (Ptch) present in the cytoplasmic membrane of the receiving cell. Binding of Shh to Ptch releases the repression that Ptch exerts on Smoothened, a seven-transmembrane-span receptor like protein essential for the transduction of Hedgehog signaling. Smoothened facilitates the interaction of different Hedgehog downstream effectors in the primary cilia, resulting in the activation of the transcription factors Gli [9]. In humans, the three Gli zinc-finger proteins (Gli1, Gli2 and Gli3) orchestrate Hedgehog-specific response in the cell by modulating gene expression. Genes of the Hedgehog pathway itself including Gli1 and Ptch1 are targets of Gli, representing a feedback loop that serve as readout of Hedgehog activity [10]. Activation of human canonical Hh pathway depends on the expression of Ptch receptors (Ptch1, Ptch2) and the decoy receptor Hhip (Hedgehog-interacting protein) [11]. Intracellular proteins that regulate Gli stability, like SUFU (Suppressor of Fused) and SPOP (speckle-type POZ protein) play also an important role in determining Gli activity and thus activation of canonical Hedgehog pathway [12]. In the past years, studies have highlighted the existence of a non-canonical Hedgehog pathway that does not require the complete Shh-Ptch-SMO-Gli axis. A non-canonical Hedgehog signaling dependent on SMO but independent of Gli, that regulate tubulogenesis and apoptosis, has been described in endothelial cells [13].With time, Gli transcription factors appear as an integrative platform of numerous signaling inputs, establishing a second kind of non-canonical Hedgehog signaling, dependent of Gli but independent of SMO. This is the case of pancreatic ductal adenocarcinoma, where Gli transcription is regulated by TGF-ß and K-ras [14].

The Hedgehog signaling pathway plays a critical role during vertebrate development controlling cell growth, survival, fate and pattern of the body plan. Alterations in Shh pathway during lung development affect epithelial/mesenchymal interactions and result in branching morphogenesis defects, impairing lung function. While Shh signaling is essential for lung development, the role that this signaling pathway can play in adult lungs remained unclear and is just now beginning to be investigated. Recent studies have highlighted the importance of Hedgehog signaling in Idiopathic pulmonary fibrosis, a lethal disease of unknown ethiology. Bolaños et al have reported that different components of the Hedgehog pathway are overexpressed in IPF lungs and IPF fibroblasts. Furthermore, fibroblasts from IPF lungs were found to respond to Shh and this response correlated with fibroblast activation *in* *vitro* [15]. An independent study conducted by Cigna et al [16] showed that primary human fibroblasts from normal and IPF lung express the main components of Hedgehog signaling pathway, associated with cell proliferation and the expression of myofibroblastic markers in fibroblasts. Whereas these studies bring out the importance of Hedgehog signaling in pulmonary fibrosis, the role of this pathway in different forms of lung cancer remains unclear. Lung cancers are classified according to histological type: the two most prevalent types of lung cancer are non-small-cell lung carcinoma (NSCLC) and small-cell-carcinoma (SCLC). NSCLC and SCLC not only exhibit differences in size and appearance of cancer cells, but also different prognosis and clinical management. Due to the heterogeneity of each lung cancer subtype, additional histological and molecular characterization is needed to select a more specific treatment. Traditionally, NSCLC were screened and treated for EGFR and K-*ras* mutations. However, the appearance of resistance to these treatments and the description of new molecular signatures have highlighted the need of a better tumor molecular-profile-directed therapy. Some studies have described the association between new patterns of gene expression in specific subsets of NSCLC [17], [18], and others suggest the application of new tumor molecular profiling in future therapies. For instance, evaluation of MMP2 and b-catenin expression has been proposed to have a potential in diagnosis of NSCLC with different clinicopathologic characteristics [19]. In SCLC, a tumor with primitive neuroendocrine features, Shh was found to be activated in neuroendocrine progenitor cells and in tumoral cells in mice [20], [21]. While these studies support a juxtacrine/autocrine activation of Hh in SCLC, it is not clear how this pathway is activated in NSCLC, the most prevalent subtype of lung cancer. Given the clinical and biological differences between NSCLC and SCLC, we hypothesized that the mechanism of activation of Shh in NSCLC is different from SCLC. We aimed to investigate the activity of Shh signaling in NSCLC in both cancer and stromal cells. In order to assess the importance of Shh signaling in NSCLC cells, we have performed knockdown of the three Gli transcription factors (Gli1-3), which mediate intracellular Shh signaling, and studied the impact thereof on NSCLC proliferation. Furthermore, we have evaluated how NSCLC cells and lung fibroblasts responded to exogenous Shh in terms of proliferation, cell migration, invasion, and extracellular matrix remodelling.

## Results

### Cyclopamine, a Hedgehog Inhibitor, Reduces NSCLC Proliferation and Viability

**Figure 1:**
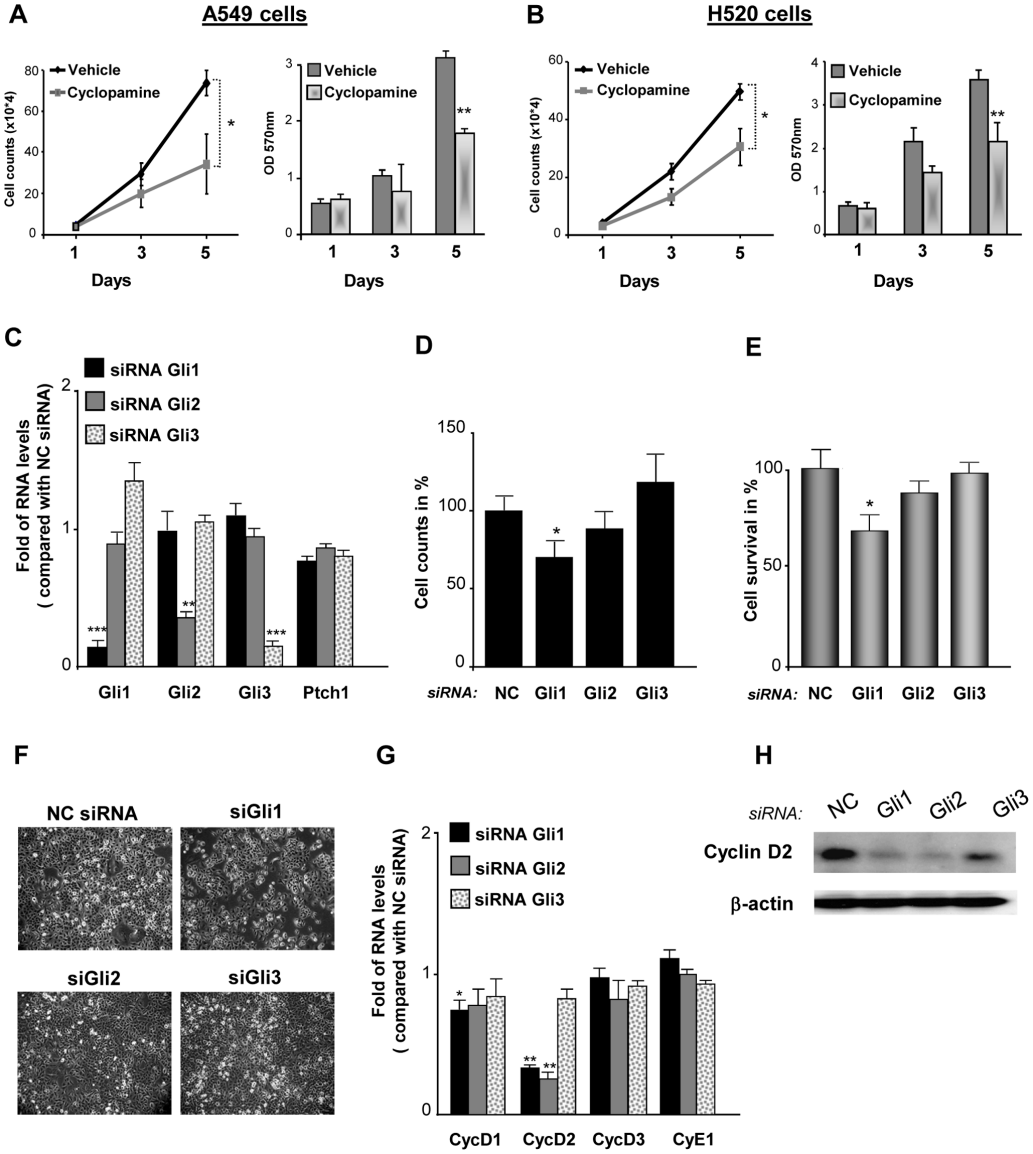
Inhibition of Hedgehog signaling reduces the proliferation of NSCLC cells. Lung adenocarcinoma A549 cells (**A**) and lung squamous carcinoma H520 cells (**B)** were cultured in presence or absence of 10 µM cyclopamine for 5 days. Proliferation was assessed by cell counting and cell survival by MTT assay. *p<0,1; **p<0,05. (**C**) The Hedgehog-responsive transcription factors Gli1, Gli2 or Gli3 were knocked down with siRNA in A549 cells. RT-qPCR was performed to confirm the specific silencing of each Gli and to assess the expression of the Hedgehog receptor Ptch1. Results are presented as fold differences of mRNA levels (2^∧∧^Ct) compared with cells transfected with a negative control siRNA (NC siRNA) having no homology in vertebrate transcriptome. **p<0,05, ***p<0,01. The impact of silencing Gli1, Gli2 or Gli3 in A549 cell proliferation was assessed by cell counting (**D**) and in cell survival by MTT assay (**E**)**.** Results are presented in percentage as relative proliferation and relative survival compared with cells transfected with the negative control siRNA (NC). *p<0,1**.** (**F**) Representative phase-contrast microscopic pictures after 72 hours of siRNA are presented**.** (**G**) RT-qPCR was performed to evaluate the effect of the siRNA of Gli1, Gli2 and Gli3 in the expression of the G1/S phase cyclins D (Cyc D1, Cyc D2, Cyc D3) and cyclin E (Cyc E1)**.** Results are presented as fold of mRNA levels (2^∧∧^Ct) compared with cells transfected with a negative control siRNA (NC siRNA) having no homology in vertebrate transcriptome. *p<0,1; **p<0,05. (**H**) Western blot of cyclin D2 in A549 cells transfected with Gli siRNA or with a negative control siRNA (NC). Blotting of ß-actin was used as loading control.

One of the most adverse characteristics of cancers is the deregulation in cell proliferation. We have therefore studied the role that Shh could have in NSCLC proliferation, using cells from adenocarcinoma and cells from squamous carcinoma, the first and second most frequent type of lung cancer respectively. Initially, we used Cyclopamine, a plant-derived steroidal alkaloid that inhibits Smoothened (SMO), a G protein-coupled receptor that transduces Shh signal in the cell, to block Shh pathway in NSCLC cells. When treated with cyclopamine, A549 adenocarcinoma cells and H520 squamous cell lung carcinoma showed a significant decrease in cell number especially at longer time points (Figures 1A and B). Cyclopamine also reduced cell survival (metabolic activity assessed by MTT assay) (Figures 1A and B) and this effect was more important with increasing doses of the inhibitor (Figure S1A and Figure S3E). To rule out that cyclopamine did not provoke a cytotoxic non-specific effect on NSCLC cells, apoptosis was determined upon cyclopamine treatment. Although cyclopamine induced a slight increase in the extent of apoptotic cells, the proportion of apoptotic cells was not statistically different between treated-cells and non-treated cells (Figure S1B and Figure S3F). In order to confirm the specific effect of cyclopamine on NSCLC proliferation and viability, SMO silencing was performed. SMO knockdown induced a decrease in both A549 and H520 cell proliferation and viability (data not shown). Altogether, these results show that blockage of Hedgehog pathway through SMO inhibition, reduces NSCLC proliferation and viability.

### Silencing of Gli1 Decreases NSCLC Proliferation by Modulating Cyclin D Expression

Although Cyclopamine has been found to impact cell proliferation in other types of cancer cells, the specific mechanism whereby Shh signaling regulates NSCLC cell cancer proliferation remains elusive. For instance, it is not known how each of the 3 human Shh-transcription factors Gli contributes to NSCLC proliferation. In order to address this question, we used small interference RNAs (siRNA) for silencing Gli1, Gli2 and Gli3. Upon a specific and important reduction in the mRNA levels of Gli1 that did not affect either Gli2 or Gli3 mRNA levels (Figure 1C), cell proliferation and cell viability was decreased in A549 adenocarcinoma cells (Figure 1D–F). Of notice, the silencing of Gli1 provoked a reduction in Ptch1 mRNA levels (Figure 1C). Because the transcription of Ptch1 depends on Gli1, the reduction of Ptch1 mRNA levels serves as an additional control indicating that the silencing of Gli1 was biologically effective. The specific silencing of Gli2, that reduced Gli2 mRNA levels and did not decrease either Gli1 or Gli3 mRNA levels (Figure 1C), diminished slightly A549 cell number and cell viability, although not in a statistically significant manner (Figure 1D and E). Finally, the siRNA of Gli3 that provoked an important diminution in Gli3 mRNA levels but not a decrease in Gli1 or Gli2 mRNA levels (Figure 1C), did not reduce A549 adenocarcinoma cell proliferation or cell viability (Figure 1D and E) and instead caused a slight increase in cell number of A549 adenocarcinoma cells (Figure 1D) along with an increase in Gli1 mRNA levels (Figure 1C). In H520 squamous lung carcinoma cells (Figure S3) and in large cell carcinoma cells (data not shown), the specific silencing of Gli1, Gli2 or Gli3 had a similar effect in cell proliferation and cyclin expression. Importantly, the expression of Shh-related genes and cyclins upon Gli1, Gli2 and Gli3 silencing was not due to Hprt1 expression because a similar pattern of expression was found when 3 independent reference genes were used in A549 (Figure S2) and in H520 cells (Figure S4).

In order to explore how the silencing of Shh pathway impacts lung adenocarcinoma proliferation, we have evaluated the changes in the expression of the cyclins D and E involved in G1/S cell cycle transition, upon Gli down-regulation. Of interest, the siRNA of Gli1 and Gli2 provoked an important decrease in cyclin D2 and a slight reduction in Cyclin D1 mRNA levels (Figure 1G). The decrease in cyclin D2 expression upon Gli1 and Gli2 knockdown was observed at the mRNA but also at the protein level (Figure 1H). Considering that Gli2 could affect cyclin D expression, we hypothesized that in combination with Gli1, this transcription factor could regulate cell proliferation in a significant manner. In order to confirm this hypothesis, we have realized the double silencing of Gli transcription factors. While the single silencing of Gli2 did not decrease significantly cell viability, the double silencing of Gli1 and Gli2 did (Figure S1C). In H520 lung squamous carcinoma cells, the specific knockdown of Gli1 and Gli2 by siRNA was found to decrease in a similar manner cyclin D1 and cyclin D2 expression (Figure S3A). Taken together, these results indicate that the blockade of Shh signaling pathway, either with cyclopamine or interfering with the transcription factor Gli1 decreases NSCLC proliferation.

### NSCLC Cells do not Activate Shh Pathway Upon Exogenous Shh Treatment

**Figure 2:**
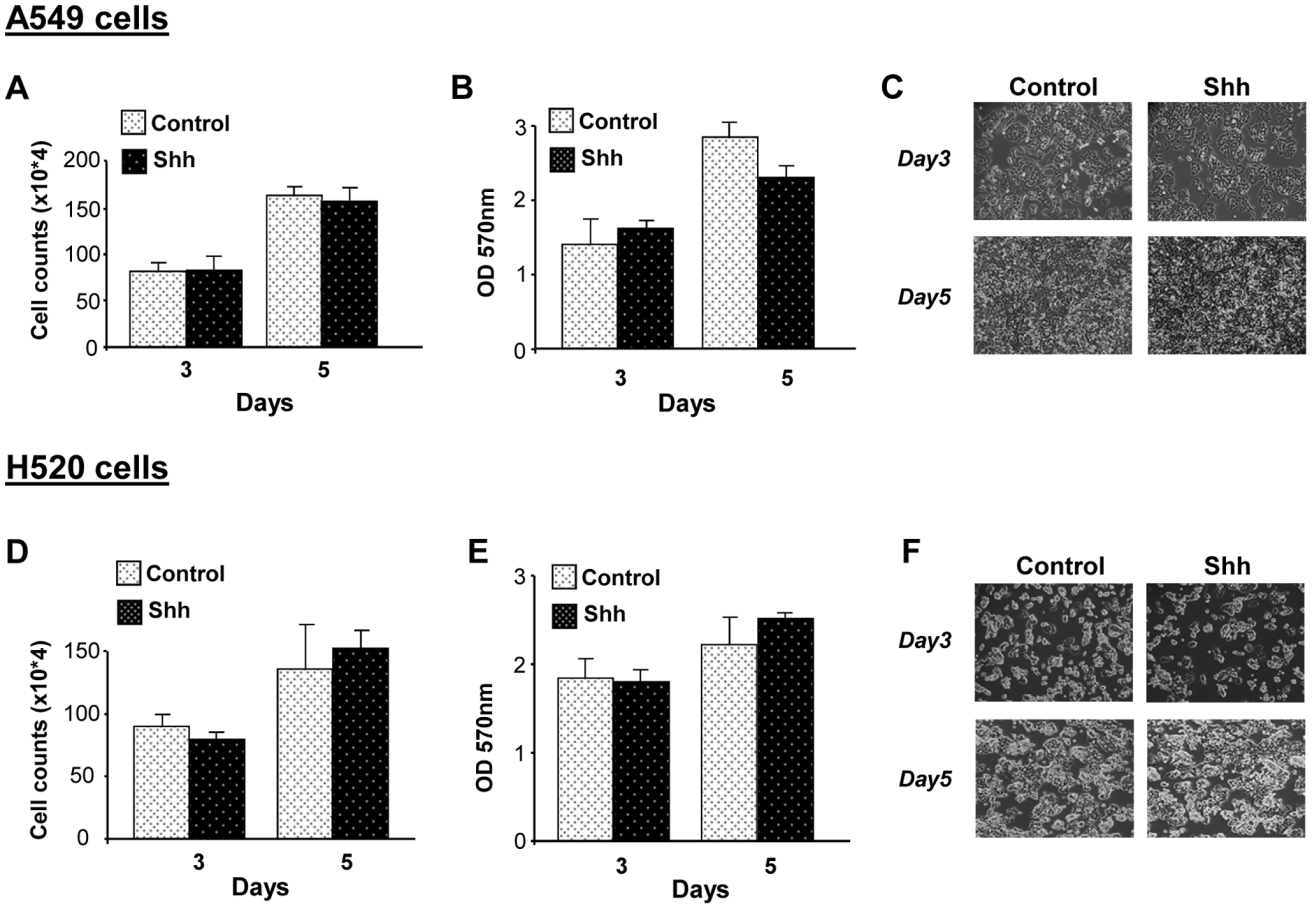
Exogenous Shh does not affect NSCLC cell proliferation. Lung adenocarcinoma A549 cells (**A–C**) and lung squamous H520 carcinoma cells (**D–F**) were treated or not with recombinant Shh (500 ng/ml). Cell proliferation was assessed by cell counting (**A, D**) and cell survival by MTT assay (**B, E**) at the indicated times. Representative phase-contrast microscopic pictures of A549 cells (**C**) and of H520 cells (**F**) are shown.

**Figure 3:**
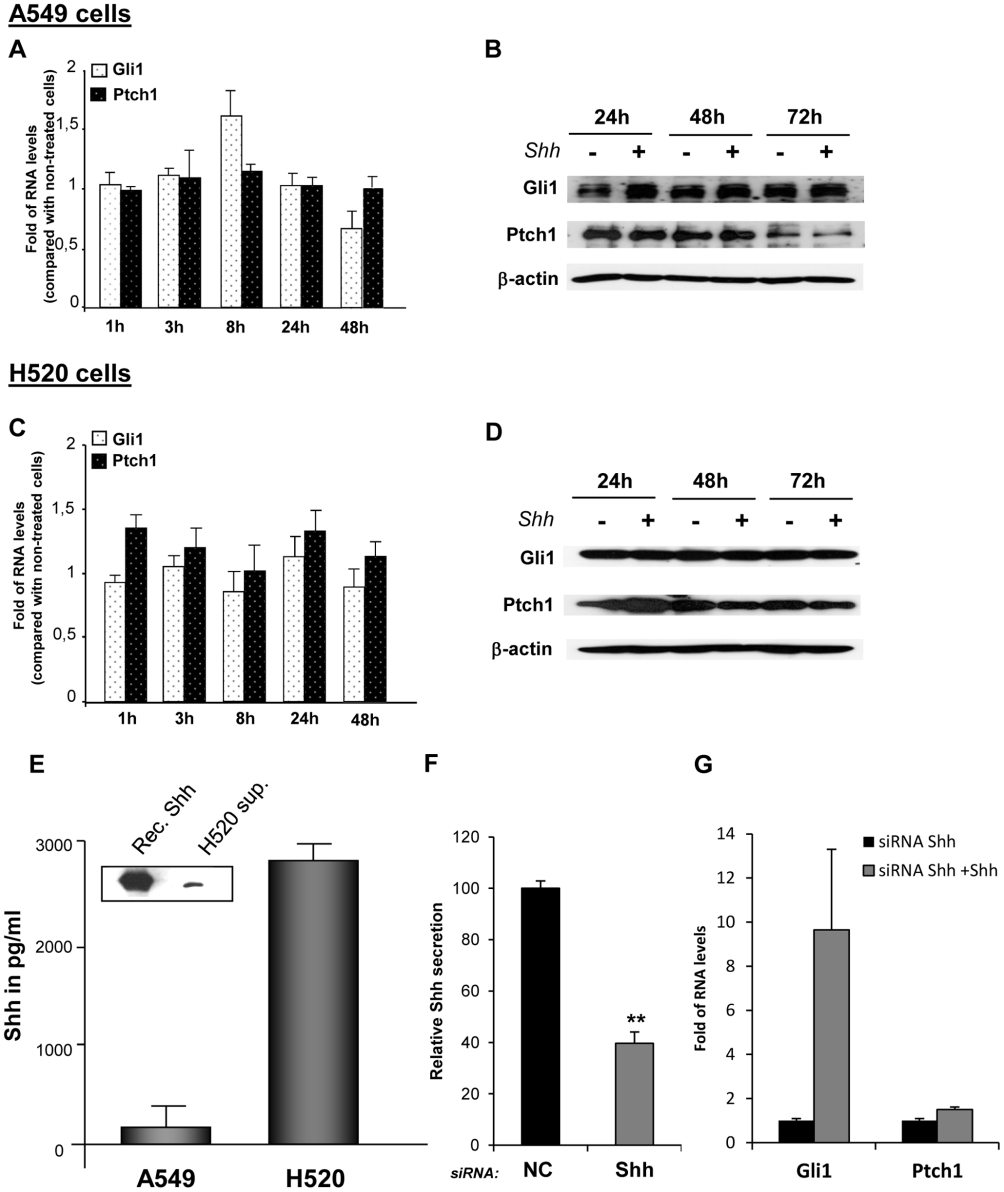
NSCLC cells do not respond to exogenous Shh but can secrete Shh ligand. NSCLC cells were treated or not with recombinant Shh (500 ng/ml) at the indicated times. RT-qPCR was performed to evaluate Gli1 and Ptch1 mRNA levels upon treatment in A549 cells (**A**) and H520 cells (**C**). Results are presented as fold differences of mRNA levels (2^∧∧^Ct) compared with non-treated cells for each time point. Western blot was performed to evaluate Gli1 and Ptch1 protein levels in A549 cells (**B**) and in H520 cells (**D**) treated or not with Shh. ß-actin was used as a loading control. Secretion of human Shh was evaluated in the supernatants of A549 and H520 cells by ELISA (**E**) and confirmed by western blot using an antibody recognizing the secreted active form of Shh (**inset E**). The western blot was performed with H520 supernatant (H520 sup.) and with recombinant Shh (Rec. Shh) used as a positive control. **(F**) The knockdown of Shh gene by siRNA was realized in H520 cells. Shh secretion was evaluated by ELISA and expressed in percentage as relative secretion compared with cells transfected with a negative control siRNA (NC) having no homology in vertebrate transcriptome. **p<0,05 (G) After silencing of Shh, NSCLC cells were treated or not with recombinant Shh (500 ng/ml). RT-qPCR was performed to evaluate Gli and Ptch1 mRNA levels. Results are presented as fold of mRNA differences (2^∧∧^Ct) in treated cells compared with non-treated cells.

Since the blockade of Shh pathway decreases NSCLC proliferation, we investigated whether exogenous Shh produces an increment in cell proliferation in these cells. Lung adenocarcinoma A549 cells and H520 squamous carcinoma cells treated with recombinant human Shh did not show a change either in cell number (Figures 2A and D) or in cell survival (Figures 2B and E). In addition, cells did not present any morphological change upon treatment (Figures 2C and F). These results suggested that NSCLC cells do not respond to Shh. In order to validate Shh treatment, we used primary embryo mouse cells from the limb buds, tissue that has been shown to be responsive to Shh. Upon Shh treatment, these cells exhibited a considerable increase in Gli1 and Ptch1 mRNA levels (60 fold and 9-fold increase respectively compared with non-treated cells; Figure S5). When A549 cells were treated with Shh, a very modest increase (1,65 fold) in Gli1 mRNA levels at 8 hours and of Gli1 protein levels at 24 hours was detected. However, at longer time points there were was no significant changes in either Gli1 or Ptch1 expression (Figures 3A and B). For H520 cells, any significant change in either Gli1 or Ptch1 at the mRNA and protein level was observed (Figures 3C and D). These results indicate that, *in vitro*, both types of NSCLC cells, compared with known Shh-responsive cells, do not notably respond to exogenous Shh.

### NSCLC Cells Secrete Shh Ligand

Using a specific ELISA test for human Shh, we found that A549 adenocarcinoma cells and more strongly H520 squamous carcinoma produce Shh (170 and 2800 pg/ml respectively, Figure 3E). This secretion can be associated with endogenous Shh production by these NSCLC cells because the concentration of Shh in the medium of each cell type was very low, comparable to the background (water).

In order to corroborate these results and to investigate if these NSCLC cells also produce the other forms of Hedgehog ligand, we have evaluated the expression of Sonic, Desert (Dhh) and Indian Hedgehog (Ihh) in A549 and H520 cells. Although we could not detect by RT-qPCR Dhh and Ihh, we found that Sonic Hedgehog was expressed in A549 and H520 cells, being more expressed in these latter (data not shown). Based on these findings, we have thus concentrated on Shh throughout our study. In addition, because H520 cells produce and secrete considerable amounts of Shh ligand, we decided to focus on these NSCLC cells for further studies related with Shh secretion. To assess more accurately the presence of the active form of Shh in H520 supernatant, western blot was performed with an antibody recognizing the processed active form of Shh (N-Shh). The presence of the N-terminal secreted peptide Shh in the supernatant of H520 cells was thereby confirmed (inset Figure 3E).

Because H520 cells secrete a considerable amount of Shh but do not respond to exogenous Shh, we aimed to investigate if this lack of response was associated with saturation of endogenous Hedgehog activity in these cells. For this, we have performed the silencing of Shh gene in H520 cells. Upon the knockdown of Shh, that reduced by 70% the secretion of Shh in H520 cells (Figure 3F), exogenous Shh increased Gli1 mRNA levels in these cells (Figure 3G). This increase, as well as a slight increment in Ptch1 mRNA levels (Figure 3G) indicate that H520 cells can respond to Shh when their endogenous levels of Shh are decreased.

### Lung Fibroblasts Strongly Respond to Exogenous Shh

**Figure 4:**
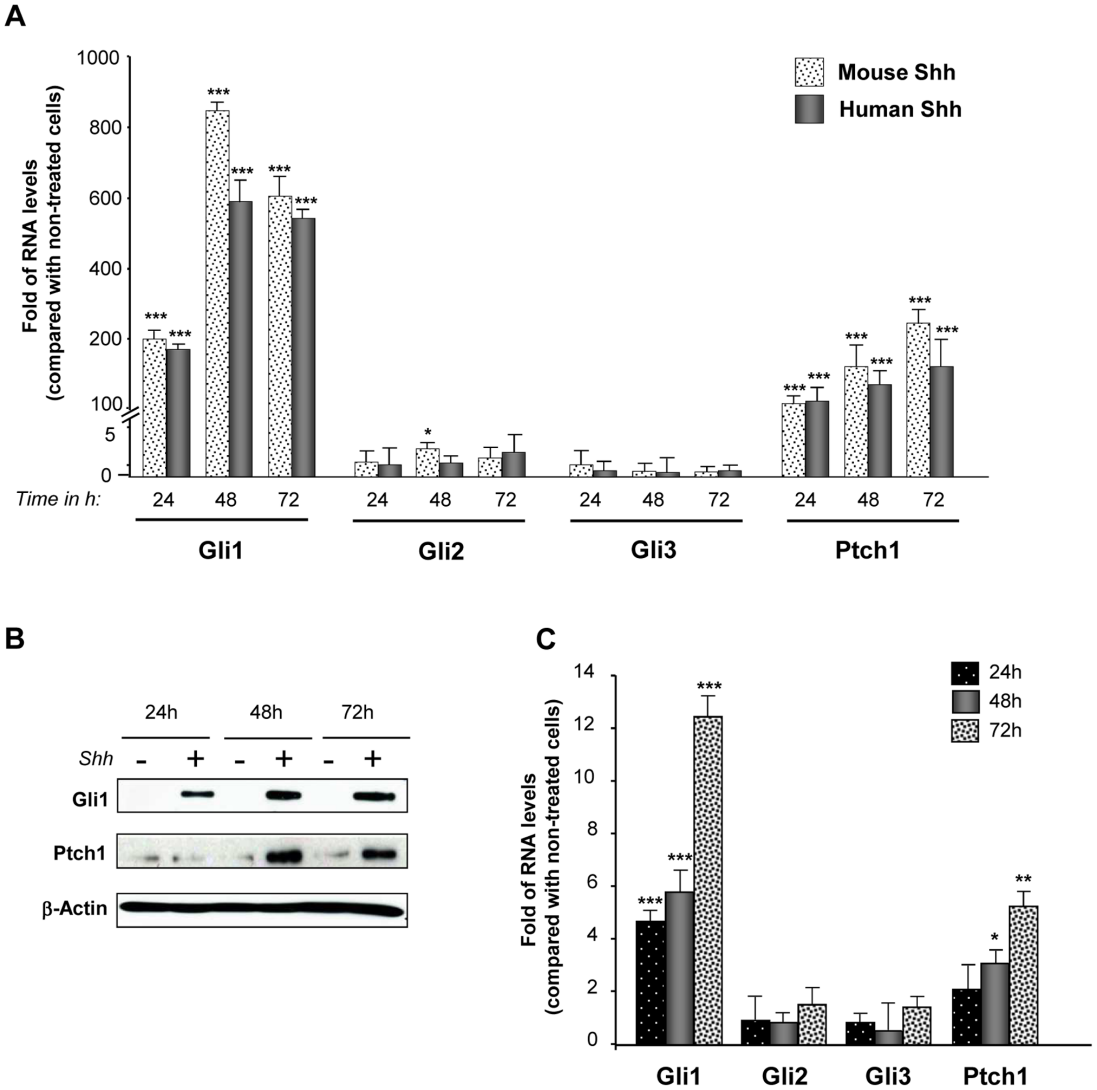
Lung fibroblasts respond to Shh treatment. Mouse lung fibroblasts CCL206 were treated or not with 500 ng/ml of recombinant mouse Shh or 500 ng/ml of human Shh for 24, 48 and 72 hours. (**A**) mRNA levels of Gli1, Gli2, Gli3 and Ptch1 upon treatment were assessed by RT-qPCR. Results are presented as fold of differences in mRNA levels (2^∧∧^Ct) of treated cells compared with non-treated cells for each time point. *p<0,1; ***p<0,01**.** (**B**) Western blot was performed to evaluate changes in Gli1 and Ptch1 protein levels in CCL206 fibroblasts treated or not with mouse Shh (500 ng/ml). ß-actin was used as a loading control. (**C**) Primary human fibroblasts were treated or not with recombinant human Shh (500 ng/ml) for 24, 48 and 72 hours. RT-qPCR was performed to assess the mRNA levels of Gli1, Gli2, Gli3 and Ptch1. Results are presented as fold of mRNA differences (2^∧∧^Ct) in treated cells compared with non-treated cells for each time point. *p<0,1; **p<0,05; ***p<0,01.

Since NSCLC cells can secrete Shh ligand but they do not strongly respond to exogenous Shh, we investigated if Shh could rather activate the adjacent stromal cells. Indeed, lung fibroblasts treated with mouse Shh exhibited a strong Hedgehog pathway activation upon treatment: Gli1 mRNA levels increased up to 800 times and Ptch1 mRNA levels had a fold increase up to 250, especially at 48 and 72 hours (Figure 4A). Similar results were obtained with human Shh (Figure 4A). Shh treatment also increased Gli1 and Ptch1 at the protein level (Figure 4B). In order to know if lung human fibroblasts from the NSCLC environment could also respond to exogenous Shh, primary human lung fibroblasts were isolated from a resected lung squamous carcinoma. Interestingly, Gli1 and Ptch1 mRNA levels were also increased upon Shh treatment in these cells (Figure 4C). These results indicate that, *in vitro*, lung fibroblasts are highly Shh-responsive cells, in contrast to NSCLC epithelial cells.

In order to investigate if the lack of response to exogenous Shh in NSCLC cells was due to an improper reception of Shh ligand in these cells, we have evaluated the expression of the receptors Ptch1, Ptch2 and of Hhip, considered as a decoy receptor, in A549, H520 cells and CLL206 fibroblasts. Ptch1 expression was found to be higher than Hhip in both NSCLC cell types (Figure S6). In addition, the relative expression of Ptch1 was higher in A549 and in H520 cells than in CCL206 fibroblasts while the relative expression of the decoy receptor Hhip was more important in lung fibroblasts than in the NSCLC cells (Figure S6). Thus, the balance between Ptch and Hhip expression does not appear to be related (at the mRNA level) with the non-responsiveness of NSCLC to exogenous Shh. As intracellular proteins such as Sufu and Spop regulate in a positive and in a negative form Gli stability respectively, we have then evaluated if an imbalance between these Gli regulators could account for the non-responsive of NSCLC to exogenous Shh. We did not find differences in the relative expression of Sufu compared with the expression of Spop for a same cell type (Figure S6). Finally, we have evaluated if the relative expression of Hh receptors and Gli regulators were different between NSCLC and lung fibroblasts upon Shh treatment. No differences were found in the expression of Ptch1, Ptch2, Hhip, Sufu and Spop upon Shh treatment, at short (1, 3, 8 h) or longer time points (24, 48 h) (data not shown).

### Lung Fibroblasts Respond to Shh Secreted by NSCLC H520 Cells

**Figure 5:**
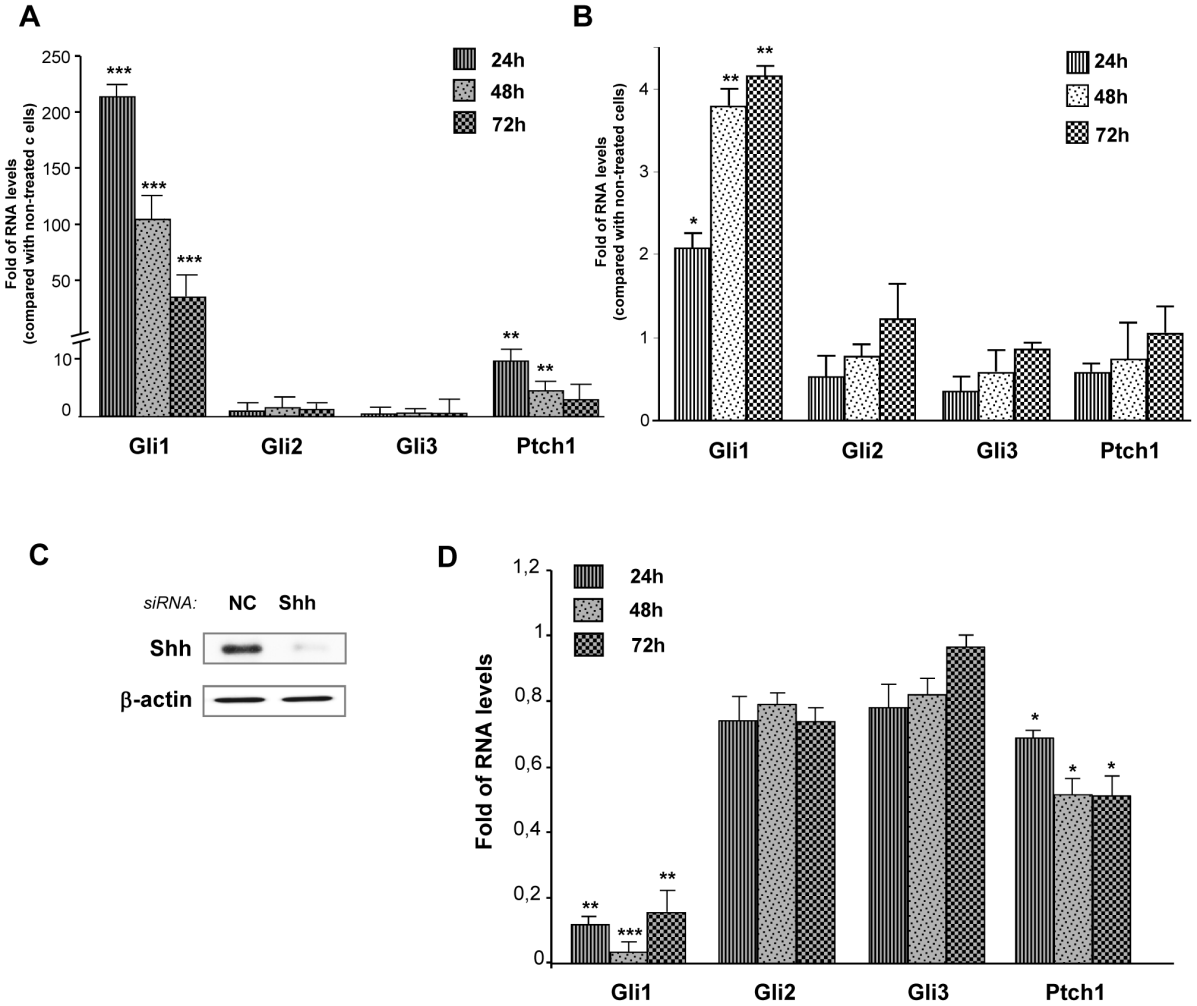
The supernatant of H520 cells activates Hedgehog pathway in lung fibroblasts . Lung fibroblasts were serum-starved for 24 hours and then treated or not with the supernatant of H520 cells for 24, 48 or 72 hours. (**A**) RT-qPCR was performed to analyze Gli1, Gli2, Gli3 and Ptch1 mRNA levels in CCL206 treated with H520 supernatant. Results are presented as fold of RNA levels in treated cells compared with non-treated cells for each time point. **p<0,05; ***p<0,01. (B**)** Primary human fibroblasts from lung squamous carcinoma were serum-starved for 24 hours and treated or not with H520 supernatant for the indicated times. RT-qPCR was performed to assess Gli1, Gli2, Gli3 and Ptch1 mRNA levels. Results are presented as fold of mRNA levels in treated cells compared with non-treated cells for each time point. *p<0,1, **p<0,05**.** (**C**) The knockdown of Shh was performed in H520 cells with siRNA. The efficiency of Shh knockdown in H520 cells was confirmed by western blot realized with the supernatant of H520 cells transfected with negative control siRNA (NC) or with the siRNA of Shh. (**D**) CCL206 fibroblasts were treated for 24, 48 and 72 hours with the supernatant of either H520 cells transfected with a negative control siRNA or with the supernatant of H520 cells transfected with the siRNA of Shh. RT-qPCR was performed to evaluate changes in Gli1, Gli2, Gli3 and Ptch1 mRNA levels. Results are presented as fold of mRNA levels (2^∧∧^Ct) in CCL206 cells treated with the supernatant of H520 transfected with the siRNA of Shh compared with fibroblasts treated with the supernatant of H520 transfected with the negative control siRNA. *p<0,1; **p<0,05; ***p<0,01.

To test whether Shh secreted by H520 squamous cells was bioactive, lung fibroblasts were treated with H520 supernatant. This resulted in the increase in Gli1 and Ptch1 mRNA levels in mouse newborn (Figure 5A) and in adult human lung fibroblasts (Figure 5B). In order to evaluate if this response was mediated by Shh secreted by H520 cells and present in the supernatant, siRNA of Shh was performed in H520 cells. This importantly decreased Shh mRNA amounts and the presence of N-Shh in the supernatant (Figure 5C) and moreover, reduced by 90% the levels of Gli1 and by 50% the levels of Ptch1 in the fibroblasts treated with H520 supernatant (Figure 5D).

### Shh Pathway Enhances Lung Fibroblast Proliferation, Invasion and Collagen Deposition

**Figure 6:**
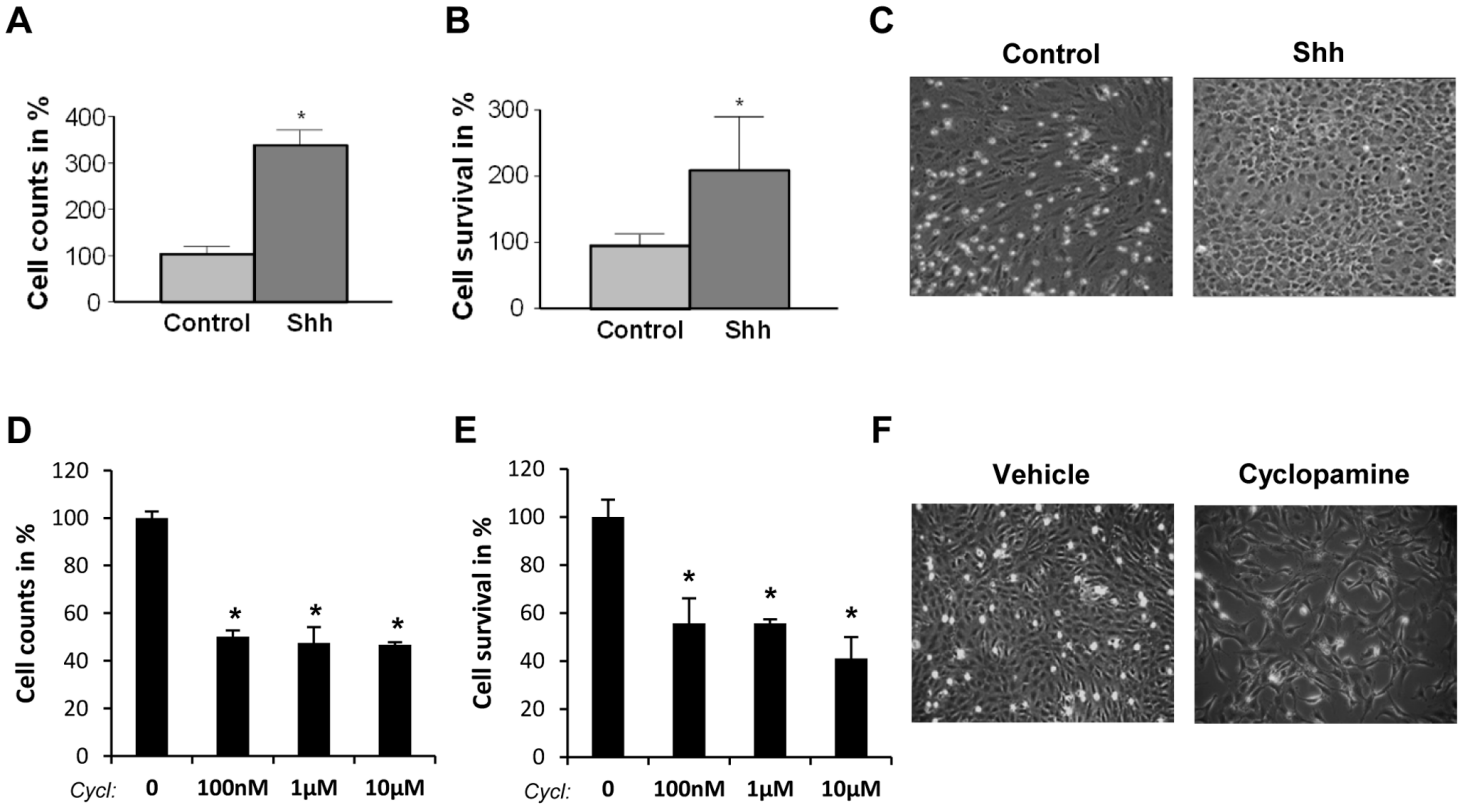
Shh pathway regulates cell proliferation and cell survival in lung fibroblasts. Proliferation of CCL206 lung fibroblasts was assessed by cell counting (**A, D**) and cell survival by MTT assay (**B, E**) after treatment with recombinant Shh (500 ng/ml) or with 100 nM, 1 µM or 10µMcyclopamine (cyclop) for 5 days. Results are presented in percentage as relative proliferation and relative survival compared to non-treated cells (**A, B**) or to vehicle (**D, E**) *p<0,1; **p<0,05. Representative phase-contrast microscope pictures upon Shh treatment (**C**) and upon 10 µM cyclopamine treatment (**F**) are shown.

**Figure 7:**
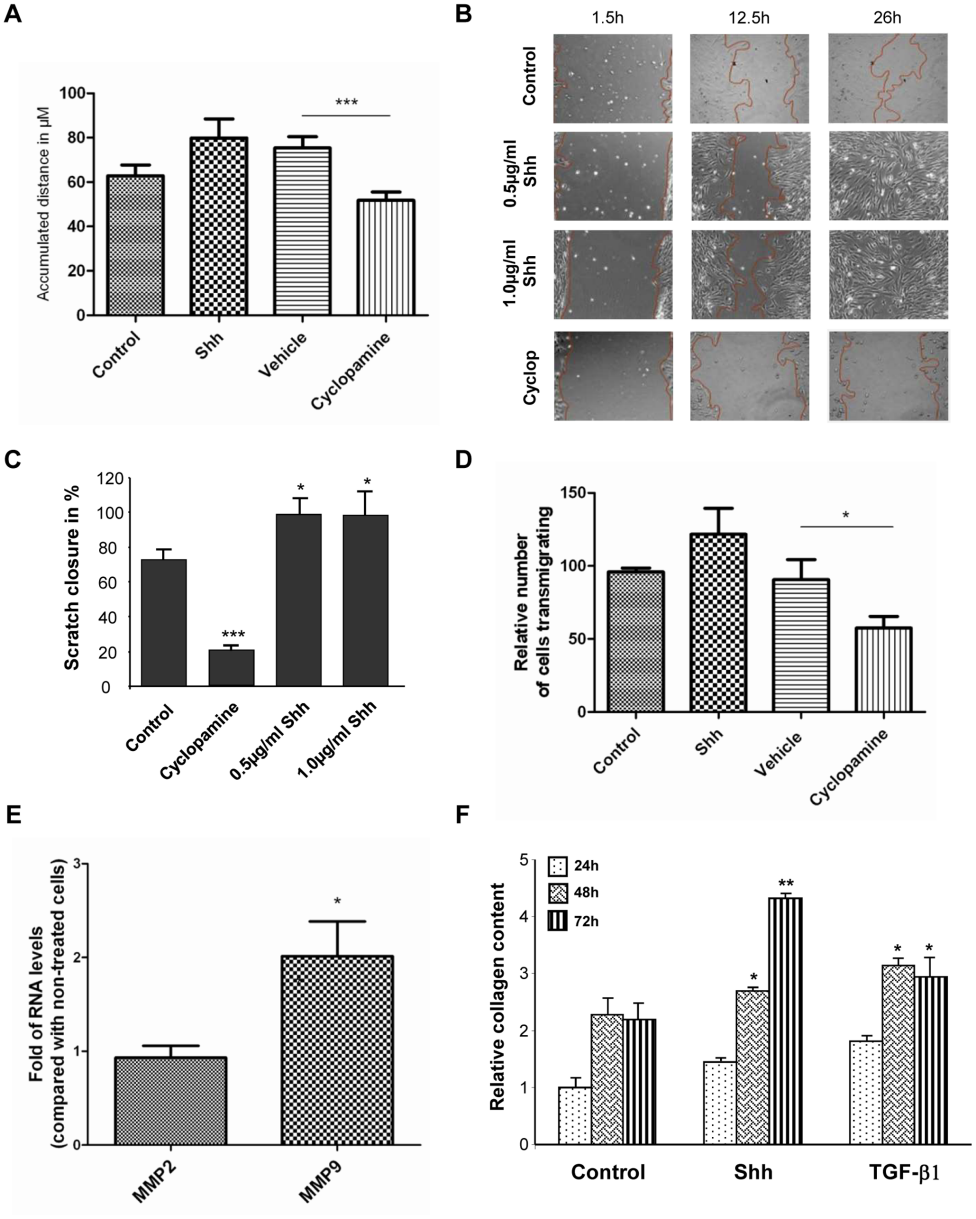
Shh pathway affects lung fibroblast migration, invasion and collagen synthesis. (**A**) Accumulated distance of migration of primary human lung fibroblasts treated with Shh (500 ng/ml) or cyclopamine (10 µM) and monitored by live cell microscopy for 48 hours. The accumulated distance of migration of each cell was determined using ImageJ. ***p<0,01. (**B**) Scratch wound assay was performed in lung fibroblasts CCL206 treated or not with Shh at the doses indicated or with 10 µM cyclopamine (cyclop) for up to 48 hours. Migration of the cells was recorded using live cell microscopy and representative pictures at 1,5, 12,5 and 26 hours are shown. The colored lines indicate the border of cell migration in each case. (**C**) The area of the wound was quantified after 26 hours and the percentage of wound closure, relative to the initial area of scratch for each case, was determined. (**D**) Transmembrane invasion assay was performed in lung fibroblasts treated with Shh (500 ng/ml) or with cyclopamine (10 µM). (**E**) RT-qPCR was performed to assess MMP2 and MMP9 expression in fibroblasts treated or not with Shh (500 ng/ml) for 48hr. Results are presented as fold of mRNA levels in treated cells compared with non-treated cells. *p<0,1. (**F**) The total collagen content of lung fibroblasts treated with Shh (500 ng/ml) or TGF-ß1 (5 ng/ml) for the indicated times was quantified using the Sircol collagen assay. *p<0,1, **p<0,05.

We have shown here that NSCLC cells do not markedly respond to exogenous Shh but can secrete bioactive Shh that induces the activation of Hedgehog signaling in lung fibroblasts. These results reveal that Shh plays an important role in mediating cancer epithelial-stromal crosstalk in NSCLC. In this setting, we have investigated the possible biological effects of Shh activation in lung fibroblasts. We have focused on different aspects of relevance in a cancer context such as proliferation, migration, invasion and extracellular matrix remodelling. While recombinant Shh increased cell survival and cell proliferation of lung fibroblasts (Figures 6A–C), cyclopamine decreased it (Figures 6D–F). This effect appear to be specifically due to the inhibition of Hedgehog pathway as cyclopamine treatment correlated with a decrease in Gli1 and Ptch1 expression in CCL206 fibroblasts (Figure S7A). Interestingly, H520 supernatant also enhanced lung fibroblast cell proliferation (Figure S7B–D). As fibroblasts have been shown to be important in facilitating cancer migration and invasion, we went on investigating the impact of Shh in these processes. Fibroblasts were treated with either Shh or with cyclopamine, and their migration was recorded up to 72 hours after treatment. Whereas Shh increased the distance of migration of fibroblasts, cyclopamine significantly decreased it (Figure 7A). In order to explore if Shh could influence fibroblast migration after an injury stimulus which may better represent the changes taking place in the tumoral tissue, we performed wound healing assays. In non-treated cells, fibroblast migrated into the wound area in a progressive manner, resulting in wound closure after 30 hours. In Shh-treated cells, this process was faster and led to wound closure after 26 hours (Figures 7B and C). On the contrary, cyclopamine decreased fibroblast migration towards the wound area and did not result in wound closure (Figures 7B and C). We then sought to investigate if Shh could affect fibroblast invasion. For this, we used transwells coated with collagen that mimics better the extracellular matrix and thus the tissue context. Cells were loaded on the top of the transwell and medium with Shh or cyclopamine was placed in the bottom, allowing the formation of a gradient. The number of cells transmigrating increased in the presence of Shh while cyclopamine decreased fibroblast invasion through the collagen coated membrane (Figure 7D). While Shh signaling regulates lung fibroblast migration and invasion, any effect was found for either Shh or cyclopamine in fibroblast adhesion assays (data not shown). Because the disorganization and changes in the architecture of the tumor microenvironment are critical hallmarks of cancer, we have investigated if activation of Shh pathway in lung fibroblasts could be associated with extracellular matrix remodelling. Exogenous Shh increased the expression of the matrix metallopeptidase MMP9 (Figure 7E), a proteolytic enzyme that plays a key role in cancer progression. Interestingly, Shh treatment also increased the synthesis of collagen in the extracellular matrix formed by fibroblast (Figure 7F). Altogether, these results indicate that Shh elicit a response in lung fibroblast that can be correlated with migration, invasion and extracellular matrix remodelling.

### Shh Mediates Bidirectional Signaling between NSCLC Cells and Lung Fibroblasts

**Figure 8:**
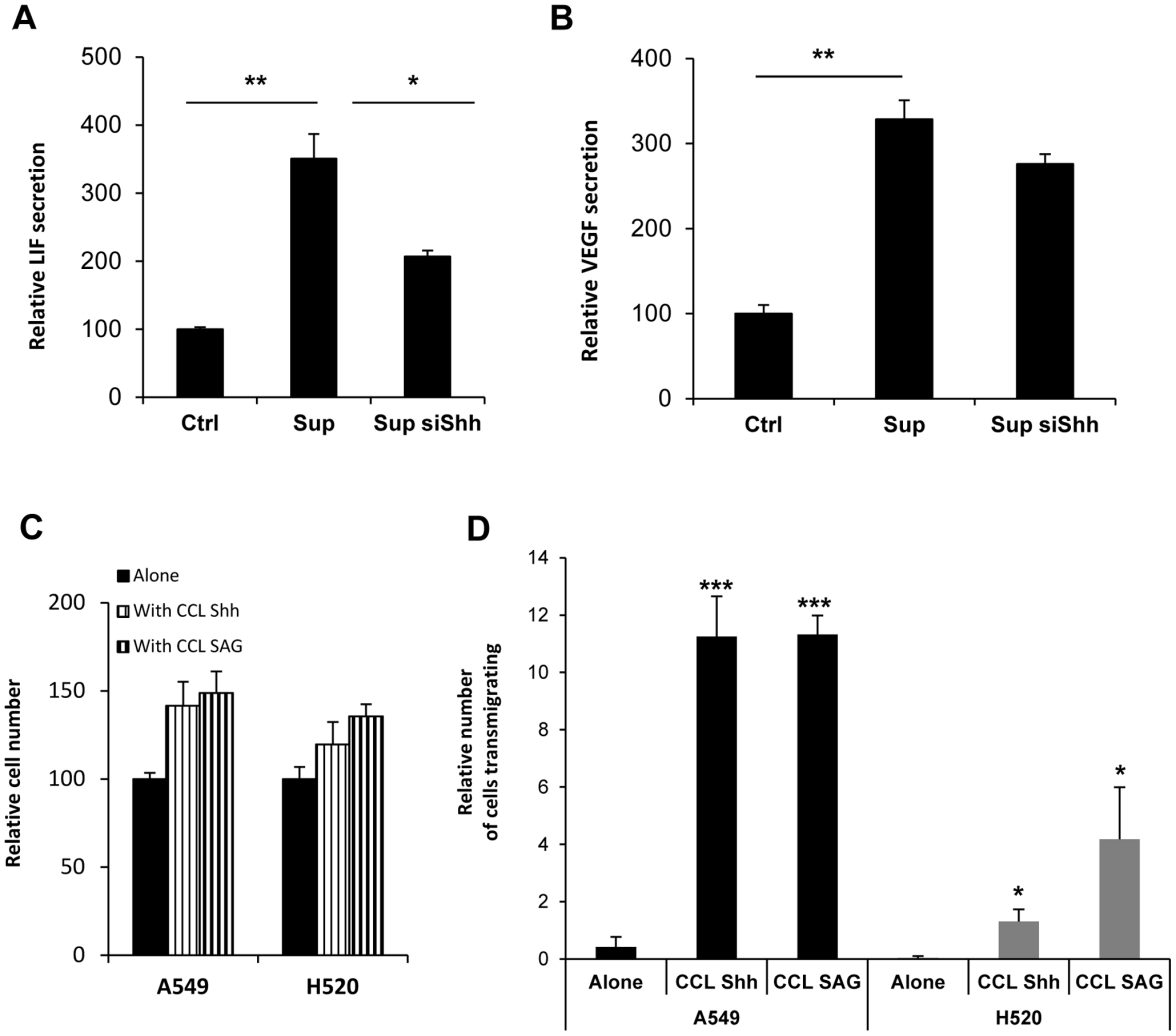
Shh mediates NSCLC/lung fibroblast reciprocal crosstalk. CCL206 lung fibroblasts were cultured or not with the supernatant of H520 transfected with a NC siRNA(Sup) or with Shh siRNA (Sup siShh) for 48 h. (A) Levels of secreted Leukemia Inhibitory Factor (LIF) were evaluated in CCL206 supernatant by multiplex biometric ELISA-based immunoassay (Bioplex system). Results are presented in percentage as relative secretion compared with cells cultured without H520 supernatant. *p<0,1; **p<0,05 (B) Levels of secreted Vascular Endothelial Growth Factor (VEGF were assessed in CCL206 supernatant by multiplex biometric ELISA-based immunoassay (Bioplex system). Results are presented in percentage as relative secretion compared with cells cultured without H520 supernatant. **p<0,05 (C) A549 and H520 cells were pre-stained with Hoechst (1 µg/ml) and then cultured for 48 h with CCL206 lung fibroblasts pre-treated with Shh (500 ng/ml) or with SAG (3 nM). The number of Hoechst positive cells was evaluated by fluorescent microscopy and is presented in percentage as relative number of cells compared with the number of cancer cells cultured alone. (D) NSCLC cells A549 and H520 were pre-stained with Hoechst (1 µg/ml) and then co-cultured for 72 h either alone or with CCL206 pre-treated with Shh (500 ng/ml) or with 3 nM SAG. The number of NSCLC cells transmigrating is expressed in percentage as the relative number of cells migrating compared with the total number of cancer cells per transwell. *p<0,1; ***p<0,01.

We have shown here that on one side NSCLC secrete bioactive Shh and, on the other side, that lung fibroblasts strongly respond to Shh. In order to further investigate how Shh could mediate epithelial- mesenchymal crosstalk in NSCLC context, we have first analyzed the impact of Shh in growth factor production. For this, we have focused on growth factors secreted by mesenchymal cells that could affect cancer progression. Among a panel of growth factors selected, three were detectable in lung fibroblasts CCL206 supernatant: Leukemia Inhibitory Factor (LIF), Vascular Endothelial Growth Factor (VEGF) and Transforming growth factor beta (TGF-ß). Interestingly, when lung fibroblasts were treated with the supernatant of H520 containing Shh, the amounts of LIF and VEGF secreted were increased (Figure 8A and 8B). To elucidate if this effect was specifically due to Shh, the knockdown of this gene was performed. The silencing of Shh in H520 cells reduced the secretion of LIF and VEGF in CCL206 fibroblasts (Figure 8A and Figure 8B). Although the supernatant of H520 also increased TGF-ß secretion in these fibroblasts, this was not changed upon Shh siRNA (Figure S8). These results indicate that Shh produced by NSCLC cells mediates in lung fibroblasts the secretion of a specific set of factors involved in cancer cell survival and cancer progression. This suggests that Shh-activated fibroblasts may in turn act on cancer epithelial cells. In order to address this point *in vitro*, lung fibroblasts were co-cultured with NSCLC cells. Interestingly, when co-cultured with Shh-treated lung fibroblasts, A549 and H520 cell proliferation was boosted and invasion was increased (Figure 8C and 8D). This effect was reproduced when fibroblasts were pre-treated with SAG, a compound that interacts with SMO and activates Shh pathway (Figure 8C and D). The results shown here indicate that NSCLC cells activate, via Shh, lung fibroblasts that in turn enhance cancer cell proliferation and invasion. Shh appear then to mediate a reciprocal crosstalk between NSCLC cells and lung fibroblasts.

## Discussion

In this study, we have shown that cyclopamine produced a progressive decrease in cell proliferation and cell viability of both, lung adenocarcinoma and lung squamous carcinoma cells. The specificity and efficacy of this inhibitor has been largely reported in different systems, where cyclopamine directly correlates with a decrease in Shh-Gli activity [20], [22], [23]. However, off-target effects of cyclopamine have been reported at high concentrations [24], [25]. The concentration needed for reaching a SMO-dependent effect may vary according to the cell type and its endogenous regulation of Hedgehog pathway. For instance, in prostate cancer cells, a significant reduction in Gli mRNA levels takes place at concentrations starting at 3 µM and this effect is stronger at 10 and 30 µM [26]. In addition, only at a concentration of 10 and 30 µM, cyclopamine reduces Gli1 at the protein level. In lung cancer cells, previous studies have reported a clear reduction in Ptch1 expression, main readout of Hedgehog activation, at 5 and 10 µM [20]. In a first approach, we have therefore used cyclopamine at 10 µM. We report here that cyclopamine decreases cell proliferation and cell viability of NSCLC cells. The specificity of this effect has been confirmed by two different approaches. On one hand, we have knocked down SMO in A549 and H520 cells. For both cell lines, the silencing of SMO decreased cell proliferation and cell viability (data not shown). On the other hand, we have performed the silencing of the Shh-specific transcription factors Gli, acting downstream of SMO. The specific knockdown of Gli1 and Gli2 decreased cell proliferation and cell viability of NSCLC cells. In addition, the fact that we did not find significant differences in cell death induced by different concentrations of cyclopamine, ruled out the possibility that cytotoxic unspecific effects of cyclopamine account for the reduction in proliferation of NSCLC cells.

Upon silencing of the three human Gli factors, Gli1 was found to be the major regulator of NSCLC cell proliferation while Gli2 had a modest effect and the silencing of Gli3 did not decrease and even slightly increased NSCLC proliferation. The fact that the silencing of Gli1 and Gli2 can decrease A549 and H520 proliferation suggests that both have redundant roles in these NSCLC cells. This is the case in mice, where the absence of Gli2 can be compensated by Gli1 [27]. If Gli1 and Gli2 can have additive effects, the specific expression and function of each one may depend on the tumoral context and in the signaling occurring in cancer cells. For instance, TGF-ß, a growth factor that plays a critical role in lung fibrosis and in cancer development, interacts with Hh pathway downstream of SMO, increasing the expression of Gli2 in mice [28], [29] and in cancer cells [30], [31]. This and other signaling crosstalk taking place in the NSCLC cells may potentiate not only Gli expression but also their effect in cancer cell proliferation. The fact that Gli1 had a stronger impact than Gli2 in NSCLC cell proliferation and survival may be due to the fact that the silencing of Gli1 was slightly more effective than the silencing of Gli2 in our study. In addition, Gli1 knockdown reduced more the expression of cyclin D1 and Cyclin D3 than the silencing of Gli2. Lastly, the role of Gli1 in regulating NSCLC proliferation may be related with the fact that Gli1 acts mainly as an activator of transcription while Gli2 can act as an activator but also as a transcription repressor [10], [32].

Since cell proliferation depends on cell cycle, we investigated if the transcription factors Gli could affect the expression of Cyclins D and E, key cyclins regulating G1/S transition. We have shown here that upon a specific knockdown of each of the transcription factors Gli, the silencing of Gli1 or Gli2 but not Gli3 decreased cyclin D1 and cyclin D2 expression. The presence of consensus Gli DNA-binding sequences in the sequence of Cyclin D1 and cyclin D2 genes, and the fact that Gli1 binds to cyclin D2 promoter [33], suggest that Gli1 and Gli2 modulate NSCLC proliferation by directly regulating cyclin D expression. Although the different isoforms of cyclins D can regulate the cell cycle in a similar way, we have found that the silencing of Gli1 and Gli2 affects in a different form the expression of each cyclin D. This might correlate with the pattern of expression of these cyclins in these cells. During organogenesis, the relative expression of each cyclin D varies according to the cell type and this might also be the case in the adult lung.

The silencing of Gli3 did not decrease either NSCLC proliferation or cyclin expression and this may be related with the repressor function of this transcription factor. In fact, a slight increase in Gli1 mRNA levels and NSCLC proliferation was observed when Gli3 was silenced. As Gli3 can be present in a truncated repressor form, the knockdown of Gli3 may have affected the formation of this repressor form, resulting in an increase in Gli1 mRNA levels and increased cell proliferation.

The fact that blocking Hh pathway in A549 and H520 cells affects NSCLC proliferation indicates that these cells have a basal level of Hedgehog activity. Indeed, we have detected Gli1 and Ptch1 proteins in both cell types in exponential growth. Hedgehog pathway in lung cancer cells may be activated upon lung epithelial injury, during the process of oncogenesis. In a naphthalene model of acute lung injury, Hh pathway was found to be activated in epithelial cells regenerating the airways [20]. Activation of other signaling pathways during epithelial transformation may impact thereafter the basal level of Hedgehog activity in lung cancer epithelial cells.

We report here that Gli1 and Ptch1 expression, main readouts of Hedgehog activity, were not significantly and long-lasting changed in NSCLC cells treated with exogenous Shh. The fact that A549 and H520 secrete different amounts of Shh and have different basal Gli1 levels suggest that Shh secretion could correlate with basal Hedgehog activity in these cells. This appears to be the case in H520 cells that exhibit a stronger response to exogenous Shh upon Shh knockdown. Thus, according to the levels of endogenous Shh in NSCLC cells, Shh pathway may be in a saturated state that does not allow detecting an additional activation of the pathway in the presence of exogenous Shh. In human tissues, the pattern of expression of Hedgehog-related genes suggest that over-expression of Shh may be partially responsible for activating hedgehog signaling pathway in NSCLC [34].

While A549 and H520 non-small-cell-lung carcinoma cells do not respond to exogenous Shh, we have shown here that they secrete Shh. The secretion of Shh is more important in H520 squamous cells than in adenocarcinoma A549 cells. In patients, similar results have been reported: Shh expression has been found to be more important in lung squamous carcinoma cells than in lung adenocarcinoma cells [19]. Thus, Shh secretion may be related with the subtype of NSCLC and its intrinsic signaling. Of notice, Shh ligand has been detected, by IHC and in situ hybridization, in NSCLC cells but not in the tumor stroma of patients [34]. Similarly, in Idiopathic pulmonary fibrosis, Shh ligand is expressed in bronchiolar and alveolar epithelial cells while Ptch1 and SMO have been observed in fibroblasts and mesenchymal cells forming fibroblast foci [15]. Furthermore, lung fibroblasts from IPF lungs are highly responsive to Shh. Thus, Shh secreted by lung epithelial cells signals to the adjacent mesenchymal cells not only in the normal lung but also in pathological conditions.

Small-cell-lung-carcinoma that originates from neuroendocrine precursors may have a different Hedgehog signaling. In a Rb1-Trp53-Ptch1 lacZ/+ mouse model of SCLC, the majority of mouse SCLC show positive Hh activity *in vivo*. Interestingly, in the same study, NSCLC mouse lung tumors induced by oncogenic Kras12 were found negative for Hedgehog activity [21]. These latter results are in accordance with our findings *in vitro* and support the idea that Hedgehog signaling can be different in SCLC and NSCLC.

In NSCLC, Hedgehog signaling seems thus to reproduce the scenario of lung development, during which Shh signaling originates from the lung epithelium and signals to the adjacent lung mesenchyme, regulating epithelial and mesenchymal growth [35]. Increasing numbers of studies have highlighted the importance of tumor microenvironment in the process of tumorigenesis. In this respect the role of stromal cells, that contribute to the development of major cancer hallmarks, has been underscored. Our data brings out the role of Shh pathway in establishing a signaling crosstalk between NSCLC cells and lung fibroblasts *in vitro*. Historically, fibroblasts were thought to be passive participants in neoplastic transformation of tissues but recent data demonstrate that they exert an active role in tumors and can promote neoplastic transformation of tissues. In this context, cancer-associated fibroblasts (CAF) may play an important role in promoting NSCLC cancer progression. Here we have shown that lung fibroblasts strongly respond to Shh and that this response is associated with an increase in fibroblast proliferation and survival. Similarly, in normal and IPF fibroblasts, Shh has been found to increase fibroblast proliferation whereas cyclopamine decrease it [15], [16]. In the context of NSCLC, Shh secreted by cancer cells may therefore enhance survival of the stromal cells upon the stressed or injured conditions of the tumoral tissue. The pool of stromal cells accumulating in the tumoral tissue may in turn promote cancer progression. In the present work, we show that Shh enhances lung fibroblast migration and invasion. As enhanced migration and invasion of CAF can result in the creation of “tunnels” through which cancer cells follow [36], together with our results this suggests that Shh play a role in NSCLC invasion throughout fibroblast migration. In addition, we have shown here that the expression of MMP9, an enzyme largely associated with neoplastic progression [37], [38] and with pulmonary metastasis formation [39], is increased in fibroblasts upon Shh treatment. A Shh-dependent increment in the expression and in the activity of MMP9 has also been reported in gastric cancer cells, correlating with the invasive capacity of these cells [40]. With our data, this suggests that Shh can impact extracellular matrix remodeling through MMP produced by fibroblasts in NSCLC tissue. Moreover, we report that Shh increased collagen synthesis in fibroblasts. The fact that Shh affects collagen production in newborn lung fibroblasts but also in normal lung fibroblast and fibroblasts from IPF lungs [15] suggest that Shh may affect lung fibroblast activation in normal but also in different pathological contexts. Previous studies have established that the stroma of tumors is stiffer than normal stroma and that deregulation of collagen cross-linking and ECM stiffness plays a causative role in cancer pathogenesis [41]. Then, Shh, by inducing collagen synthesis in stromal fibroblasts, may induce the remodeling of the extracellular matrix in NSCLC tissue and therefore promote cancer progression.

Interestingly, Shh appear to modulate not only cancer to mesenchymal crosstalk but also mesenchymal to cancer signaling. Actually, we have found that NSCLC cells increase, via Shh, lung fibroblast survival and proliferation. Fibroblasts, in turn, when co-cultured with NSCLC cells, improve cancer cell proliferation and viability. Furthermore, Shh-activated fibroblasts increased the secretion of factors such as LIF and VEGF that have an important effect on cancer cells. Indeed, LIF participates in alveolar epithelium differentiation and vasculogenesis [42]. In addition, lung preneoplastic and cancer cells have been shown to respond to LIF [43], a factor involved in cancer metastasis [44]. Thus, LIF secretion by Shh-activated lung fibroblasts may enhance malignant transformation and metastatic potential in NSCLC context. On the other side, Shh-dependent VEGF up regulation may potentiate NSCLC angiogenesis as it is the case in different *in vitro* and *in vivo* models [45]–[47]. Shh-induced VEGF may induce the remodelling of pulmonary vascular network, through its effect in endothelial cells. Finally, increased LIF and VEGF secretion by Shh-activated fibroblasts may affect the stemness of cancer cells [48], [49].

Studying the epithelial/mesenchymal crosstalk mediated by Shh in NSCLC will improve our understanding of NSCLC biology and might acquire a prognostic application. For instance, in breast cancer patients, a paracrine signature defined as high epithelial Hh ligand and high stromal Gli1 has been found to be an independent predictor for overall survival in multivariate analysis in a cohort of 279 patients [50]. In the adult lung, Shh is either not detected [15], [34] either found in the alveolar and bronchiolar epithelia [16], [51]. Although these differences may be related with the use of different antibody or tissue preparations, Shh expression appear to be subtle in the normal lung tissue. Upon injury, Shh has been found to be highly expressed in the epithelial compartment of regenerating airways in the mouse [20]. Similarly, in human lungs, Shh has been found in areas of epithelium injury and repair [15], [16], [51] while nuclear Gli1 has been reported in epithelial cells, fibroblasts and inflammatory cells of fibroblastic foci in tissues from pulmonary fibrotic patients [15], [16]. In NSCLC, few studies have evaluated the expression of Shh related proteins in the tissues. By immunohistochemical analysis of 96 specimens, Chi et al. found that the expression of Shh was restricted to the tumor area and not present in the stroma, However, due to the limited number of tumors with activated hedgehog signaling in this study, no statistical analysis could be performed and data concerning patient survival was not included [34]. In a study conducted by Yuan et al. with a tumor tissue array containing 120 NSCLC samples, Gli1 was found to be expressed in the majority of lung adenocarcinoma and squamous cell carcinoma, indicating a basal Hedgehog activity in these cancer cells [23]. Nonetheless, no assessment of Gli1 expression was realized in the stromal cancer compartment and no correlation was established with patient follow-up. In the future, defining Shh and Gli1 signatures in both, cancer epithelial and stromal cells will be relevant for a more accurate and comprehensive study of Hedgehog signaling in NSCLC. Furthermore, strategies for NSCLC treatment should take into account Hedgehog activation in the cancer cells but also the stromal cells of the NSCLC tissue.

## Materials and Methods

### Ethics Statement

Resected human lung tissue was used for isolation of primary cells. Participants provided written informed consent to participate in this study, in accordance with approval by the local ethics committee of the LMU (Ludwig-Maximilians Universität) of Munich, Germany (Project 333-10).

### Cell Culture

The lung adenocarcinoma cell line A549 was purchased from the German Collection of Microorganisms and Cell Cultures DSMZ, the lung squamous carcinoma NCI-H520 (ATCC n° HTB-182) cell line and the mouse fibroblast CCL-206 (ATCC n°CCL-206) were purchased from the American Type Culture Collection ATCC. Each cell line was grown in the medium recommended by the providers. Primary mouse limb bud cells were a kind gift of Dr. Heiko Lickert (Helmholtz Zentrum Muenchen, Germany) and were cultivated in DMEM with 10% of heat inactivated foetal calf serum (Fcs) as previously described [52].

Primary human fibroblasts were isolated from lung squamous carcinoma explants. Identification of fibroblasts was based on the expression of vimentin, collagen and α-SMA and the expression of these genes was assessed at different passages.

Primary cells and cell lines were maintained in a humidified incubator in an atmosphere of 5% CO2 at 37°C.

### Cell Proliferation and Cell Survival Assessment

Cells were seeded in 6-well-plate at low density; one day after seeding, cells were treated or not with cyclopamine (LC laboratories, Woburn, MA, USA) at the indicated doses or with vehicle (ethanol). For Shh treatment, cells were serum-starved for 24 hours and then treated with recombinant mouse Shh (R&D Systems, Minneapolis, MN, USA) or recombinant human Shh (R&D Systems). Cell proliferation was assessed by counting the number of viable cells using a CASY Cell Counter and analyser system (Casy Roche Innovativs model TT, Reutlingen, Germany). Cell survival was evaluated by MTT assay (Thiazolyl Blue Tetrazolium Blue, Sigma-Aldrich, Schnelldorf, Germany). For co-culture experiments, A549 or H520 cells were pre-incubated with 1 µg/ml Hoechst (Thermo Scientific, Pierce, Bonn, Germany) for 30 min at 37°C. Cells were rinced with PBS and resuspended in DMEM:F12 medium containing 1%Fcs and then co-cultured with CCL206 fibroblasts for 72 h. Fibroblasts were previously serum-starved and treated or not with Shh (500 ng/ml) or 3 nM SAG (Calbiochem, Darmstadt, Germany). The number of Hoechst positive cells was evaluated by fluorescent microscopy using an AxioVision microscope (Carl Zeiss, Munich, Germany).

### Reverse Transcription and Quantitative Real-time PCR

RNA was extracted using NucleoSpin RNA II kit (Macherey & Nagel, Düren, Germany) according to the manufactureŕs protocol, including a digestion with RNase-free DNase. 1 µg of RNA was reverse-transcribed to cDNA using MMLV reverse transcriptase (Promega, Manheim, Germany) and random hexamers (Applied Biosystems, Darmstadt, Germany). Quantitative real-time PCR was performed on a Roche Light Cycler 480 II machine using SYBR Green PCR Master Mix (Roche, Manheim, Germany). Primers (Table S1) were designed using Nucleotide blast from National Center for Biotechnology Information (http://blast.ncbi.nlm.nih.gov). Hprt1 (hypoxanthine guanine phosphoribosyl transferase), ubiquitously and equally expressed gene free of pseudogenes was used as a reference gene in all qRT-PCR reactions. Three genes, WDR89 (WD repeat domain 89), DHX8 (DEAH (Asp-Glu-Ala-His) box polypeptide 8) and UBC (Ubiquitin C), reported to have a stable expression in a wide set of human lung neoplasm arrays [53], were used as additional reference genes to confirm the relative expression of the genes studied. Relative transcript abundance of a gene is expressed as fold of relative changes in mRNA levels compared to controls, using the 2-ΔΔCt calculations (ΔΔCt = ΔCt treated-ΔCt control).

### Western Blotting

Cells were lysed with lysis buffer (50 mM Tris/HCL pH 8, 150 mM NaCl, 1% NP-40, 0,5% Sodium Deoxycholate, 0,1% SDS) supplemented with CompleteTM Proteinase Inhibitor Cocktail (Merck Biosciences, Darmstadt, Germany). Cell lysates were resolved by SDS-PAGE, loaded in Lammli buffer (260 mM TrisHCl,40% glycerol, 8% SDS, 0.004% bromphenolblue, 5% β-mercaptoethanol), and transferred to polyvinylidene difluoride (PVDF) membrane (Amersham, Bucks, UK). Membranes were blocked with TBS containing 5% non-fat milk and 0.1%Tween and incubated with Gli1 (Cell Signaling 2643, New England Biolabs, Frankfurt, Germany), Ptch1 (Abcam ab39266, Cambridge, UK), Shh (Cell signaling 2207), cyclin D2 (Cell signaling 3741) or β-Actin-Peroxidase (Sigma A3854). Secondary antibodies coupled to horseradish peroxidase were purchased from GE Healthcare, and chemiluminescence was used to detect immunoreactive bands on autoradiography film (GE Healthcare, Uppsala, Sweden).

### RNA Interference Experiments

siRNA transfections were performed with Lipofectamine RNAiMax (Invitrogen, Karlsruhe, Germany) according to the manufacturer’s instructions. Pre-designed siRNAs against human Gli1 (s5815), Gli2 (s5817), Gli3 (s5822) and SMO (s13165) were purchased from Applied Biosystems. A negative control siRNA (Applied Biosystems AM4611) with no homology to known genes was used as an irrelevant siRNA.

### Measurement of Secreted Human Shh

The concentration of Shh in the supernatant of NSCLC cells was determined using a Sonic Hedgehog human ELISA kit (Abcam ab100639), following the manufacturer's guidelines. The cell culture medium for each cell type was used as a negative control and its value was subtracted from the values obtained for the respective cell type.

### Bioplex Assay

The supernatant of H520 cells transfected with a negative control siRNA or with Shh siRNA was obtained and used to culture CCL206 fibroblasts for 48 h. The supernatant of CCL206 cultured or not with H520 supernatants, was collected and centrifuged (1000 g, 15 min, 4°C). A multiplex biometric ELISA-based immunoassay, containing dyed microspheres conjugated with a monoclonal antibody specific for each target protein was used according to the manufacturer’s instructions (Bioplex, Bio-Rad Lab., Inc., Hercules, CA, USA). Soluble molecules were measured using the commercially available kits for mouse basic FGF, VEGF, PDGF-bb, LIF, M-CSF, G-CSF, TGF-ß1, TGF-ß2 and TGF-ß3. In each independent experiment, each sample was measured two times. Levels of growth factors and cytokines were determined using a Bio-Plex array reader (Bio-Rad). The analytes concentration was calculated using a standard curve, with software provided by the manufacturer (Bio-Plex Manager Software).

### Time Lapse Microscopy

Cells were seeded in 12-well-plate and treated with 10 µM cyclopamine in complete medium or serum-starved for 24 hours and treated or not with Shh (500 ng/ml). Cells were incubated in an Axio Observer microscope chamber equipped with an AxioCam camera (Carl Zeiss, Munich, Germany). Images were captured every 30 min for 48 hours. Images were analyzed with Axiovision 4.0 software (Carl Zeiss). Single cells were tracked and accumulated distance (µM) was calculated with the Chemotaxis Tool plug in from ImageJ analysis software.

### Wound Healing Assay

Lung fibroblasts CCL206 were seeded on 24-well-plate in complete medium until reaching 90% of confluence. Cells were then serum-starved for 24 hours in the case of Shh treatment. Monolayers were scratched with a fine pipette tip. Cells were washed once with PBS to remove detached cells and then cultivated in complete medium containing 10 µM cyclopamine or vehicle (ethanol) or in 1% Fcs medium containing or not Shh (500 ng/ml or 1000 ng/ml). Time-lapse movies were acquired over a period of 48 hours using the Axio Observer microscope equipped with an AxioCam camera (Carl Zeiss). Images were captured at 30 min intervals, and analysed with Axiovision 4.0 software (Carl Zeiss). The initial and final area of the scratch was determined and used to calculate the percentage of wound closure after 26 hours.

### Transwell Invasion Assay

Cell invasion was determined using transwells (Greiner Bio-one, Frickenhausen, Germany) with a pore size of 8 µm. Transwells were coated with 10 µ/ml of Rat Tail collagen Type I (Sigma-Aldrich). 24 hours previous to invasion assay, cells for Shh treatment were serum-starved. The next day, cells were resuspended and loaded on the transwell placed in a 24-well-plate. The bottom of the well contained complete medium and 10 µM cyclopamine or vehicle (ethanol) or medium with 0,5% of Fcs +/− Shh (500 ng/ml). Cells were incubated at 37°C in a humidified 5%CO2 atmosphere for 6 hours. Cells on the upper side of the membrane were scraped off and the cells that migrated to the lower side were fixed and stained with Crystal Violet. The number of migrated cells was counted in four-five random fields under ×20 magnification with a phase contrast microscope (Zeiss Axiovert). For co-culture experiments, A549 or H520 cells were pre-incubated with 1 µg/ml Hoechst (Thermo Scientific) for 30 min at 37°C. Cells were rinced with PBS and resuspended in DMEM:F12 medium containing 1%Fcs and co-cultured with CCL206 fibroblasts for 72 h. Fibroblasts were previously serum-starved and treated or not with Shh (500 ng/ml) or 3 nM SAG (Calbiochem).The number of transmigrated Hoechst positive cells was determined by fluorescent microscopy using an AxioVision microscope (Carl Zeiss, Munich, Germany).

### Collagen Assay

CCL206 lung fibroblasts were plated in 60 mm dishes and serum-starved for 24 h and treated for 24, 48 and 72hr with 500 ng/ml of mouse Shh or 5 ng/ml of TGF-ß1 (R&D Systems). Total collagen content was determined using the Sircol Collagen Assay kit (Biocolor, County Antrim, UK). Equal amounts of protein lysates were added to 1 ml of Sircol dye reagent; the assay was performed according to the manufacturer’s instructions. Samples and collagen standards were read at 540 nm on a Multifunctional Microplate Reader (Berthold Biotechnologies, Bad Wildbad, Germany). Collagen concentrations were calculated using a standard curve made with acid-soluble type 1 collagen.

### Flow Cytometry Assay of Apoptosis

A549 and H520 cells were cultured in the absence or presence of 100 nM, 1 µM, or 10 µM of cyclopamine for 72 h. Cells were then trypsinized, resuspended in PBS/5%Fbs and incubated with annexin V-fluorescein isothiocyanate (BD Biosciences 556419) for 30 min in the dark at room temperature. Cells were washed and incubated with propidium iodide (PI, Sigma P4864) for 10 min in the dark at room temperature. Cells were washed and flow cytometric analysis was immediately performed using a LSRII Instrument (BD Biosciences). Three population of cells were distinguished: viable cells not undergoing apoptosis (Annexin V-FITC and PI negative), cells undergoing apoptosis (Annexin V-FITC positive) and dead cells (Annexin V-FITC negative and PI positive). Unstained cells and cells incubated with suitable isotype control antibodies were used as negative control. The results were analyzed using BD FACSDiva Software.

### Statistical Analysis

Data was obtained from experiments performed at least three times and was represented as means ± SEM. Differences among groups were analyzed using unpaired t-tests or ANOVA together with a post-hoc Bonferroni analysis.

## Supporting Information

Figure S1 **Inhibition of Hedgehog signaling decreases A549 cell survival.** (**A**) Lung adenocarcinoma A549 cells were cultured in absence or presence of 100 nM, 1 µM or 10 µM of cyclopamine for 1, 3 and 5 days. Cell survival was assessed by MTT assay and is expressed in percentage relative to non-treated cells. *p<0,1. (B) The proportion of A549 apoptotic and dead cells upon 72 hours of cyclopamine treatment (100 nM, 1 µM or 10 mM) was assessed using annexin V/PI staining and flow cytometry. The percentage of alive cells, apoptotic and dead cells, from the gated population are presented. (C) A549 Cells were transfected with one or two siRNA at the same time, as indicated. Cell survival was assessed by MTT assay 72 hours after the transfection. Results are presented as relative cell survival compared with cells transfected with the negative control siRNA (NC). *p<0,1; **p<0,05.
(PDF)

Figure S2 **The pattern of expression of Shh-related genes and cyclins upon Gli knockdown in A549 cells is similar when different reference genes are used.** The knockdown of Gli1, Gli2 or Gli3 was performed in A549 cells using siRNA. The specific silencing of each human transcription factor Gli and the effect of the silencing of each Gli in the expression of Hedgehog receptor Ptch1 and in the G1/S phase cyclins D (Cyc D1, Cyc D2, Cyc D3) and cyclin E (Cyc E1) was studied by RT-qPCR. Relative transcript abundance of a gene (vertical axes) is expressed as fold of relative changes in mRNA levels (2^∧∧^Ct) compared with cells transfected with a negative control siRNA (NC siRNA) having no homology in vertebrate transcriptome. Relative mRNA levels were calculated taking four different genes for reference: Hprt1 (A), Dhx8 (B), Wdr89(C) or Ubc (D). *p<0,1; **p<0,05; ***p<0,01.
(PDF)

Figure S3 **Silencing of Gli1 decreases lung cancer squamous H520 cell proliferation, cyclin D1 and cyclin D2 expression.** The knockdown of Gli1, Gli2 or Gli3 was performed in H520 cells using siRNA. (**A**) The specific silencing of each human transcription factor Gli and the effect of the silencing of each Gli in the expression of Hedgehog receptor Ptch1 and in the G1/S phase cyclins D (Cyc D1, Cyc D2, Cyc D3) and cyclin E (Cyc E1) was studied by RT-qPCR. *p<0,1; **p<0,05; ***p<0,01. (**B**) Representative phase-contrast microscopic pictures after 72hours of siRNA are presented. The impact of silencing Gli1, Gli2 or Gli3 in H520 cell proliferation was assessed by cell counting (**C**) and in cell survival by MTT assay (**D**). Results are presented in percentage as relative proliferation and relative survival compared with cells transfected with the negative control siRNA (NC). *p<0,1. (E) H520 cells were cultured in absence or presence of 100 nM, 1 µM or 10 µM of cyclopamine for 1, 3 and 5 days. Cell survival was assessed by MTT assay and is expressed in percentage relative to non-treated cells. *p<0, 1 (F) The proportion of H520 apoptotic and dead cells upon 72 hours of cyclopamine treatment (100 nM, 1 µM or 10 mM) was assessed by using annexin V/PI staining and flow cytometry. The percentage of alive, apoptotic and dead cells, from the gated population are presented.
(PDF)

Figure S4 **The pattern of expression of Shh-related genes and cyclins upon Gli knockdown in H520 cells is similar when different reference genes are used.** The knockdown of Gli1, Gli2 or Gli3 was performed in H520 cells using siRNA. The specific silencing of each human transcription factor Gli and the effect of the silencing of each Gli in the expression of Hedgehog receptor Ptch1 and in the G1/S phase cyclins D (Cyc D1, Cyc D2, Cyc D3) and cyclin E (Cyc E1) was studied by RT-qPCR. Relative transcript abundance of a gene (vertical axes) is expressed as fold of relative changes in mRNA levels (2^∧∧^Ct) compared with cells transfected with a negative control siRNA (NC siRNA) having no homology in vertebrate transcriptome. Relative mRNA levels were calculated taking four different genes for reference: Hprt1 (A), Dhx8 (B), Wdr89(C) or Ubc (D). *p<0,1; **p<0,05; ***p<0,01.
(PDF)

Figure S5 **Mouse primary limb buds cells were used as a positive control for exogenous Shh treatment.** Primary limb buds cells from mouse embryo were serum-starved for 24 hours and then treated or not with mouse Shh (500 ng/ml) for the indicated times. Gli1 and Ptch1 mRNA levels were evaluated by RT-qPCR. Relative transcript abundance of a gene is expressed as fold of relative changes in mRNA levels (2^∧∧^Ct) compared with non-treated cells for each time point. *p<0,1; **p<0,05; ***p<0,01. *p<0,1;**p<0,05; ***p<0,01.
(PDF)

Figure S6 **The relative expression of Ptch, Hhip, Sufu and Spop in NSCLC cells and CCL206 lung fibroblasts.** The relative mRNA expression of Ptch1, Ptch2, Hhip, Sufu and Spop was assessed by RT-qPCR in cells cultured in medium containing 1% of Fcs. Relative transcript abundance of a gene is expressed as minus dCt (dCt =  Ct of gene of interest – Ct reference gene) compared with Hprt1.
(PDF)

Figure S7 **Shh pathway correlates with lung fibroblast proliferation and cell survival. (A**) Cyclopamine reduces Gli1 and Ptch1 expression in CCL206 lung fibroblasts. CCL206 fibroblasts were treated or not with 1 nM, 1 µM or 10 µM of cyclopamine for 72 h. Gli1 and Ptch1 mRNA levels were evaluated by RT-qPCR. Relative transcript abundance of each gene is expressed as fold of relative changes in mRNA levels compared to non-treated cells. *p<0,1; **p<0,05. Lung fibroblasts were cultured for 5 days with normal medium or with the supernatant of H520 cells containing 0,5% of Fcs. Cell proliferation was assessed by cell counting (**B**) and cell survival by MTT assay (**C**). Results are presented in percentage as relative proliferation and relative survival compared with control condition (fibroblasts grown in normal medium). *p<0,1; **p<0,05. (**D**) Representative phase-contrast microscope pictures of fibroblasts upon treatment are shown.
(PDF)

Figure S8 **TGF-ß secretion in lung fibroblasts is increased by NSCLC supernatant but does not depend on Shh.** CCL206 lung fibroblasts were cultured or not with the supernatant of H520 transfected with a NC siRNA(Sup) or with Shh siRNA (Sup siShh) for 48 h. Levels of secreted TGF-b1 (A) and TGF-b2 (B) were evaluated in CCL206 supernatant by multiplex biometric ELISA-based immunoassay (Bioplex system). Results are presented in percentage as relative secretion compared with cells cultured without H520 supernatant. ***p<0,01.
(PDF)

Table S1 **Primer sequences.**
(DOCX)

## References

[1] Bray F, Ren J-S, Masuyer E, Ferlay J (2013) Global estimates of cancer prevalence for 27 sites in the adult population in 2008. Int J Cancer 132: 1133–45. 22752881 10.1002/ijc.27711

[2] Trimboli AJ, Cantemir-Stone CZ, Li F, Wallace JA, Merchant A, et al (2009) Pten in stromal fibroblasts suppresses mammary epithelial tumours. Nature 461: 1084–91. 19847259 10.1038/nature08486 PMC2767301

[3] Migneco G, Whitaker-Menezes D, Chiavarina B, Castello-Cros R, Pavlides S, et al (2010) Glycolytic cancer associated fibroblasts promote breast cancer tumor growth, without a measurable increase in angiogenesis: evidence for stromal-epithelial metabolic coupling. Cell Cycle 9: 2412–22. 20562527 10.4161/cc.9.12.11989

[4] Castello-Cros R, Bonuccelli G, Molchansky A, Capozza F, Witkiewicz AK, et al (2011) Matrix remodeling stimulates stromal autophagy, «fueling» cancer cell mitochondrial metabolism and metastasis. Cell Cycle 10: 2021–34. 21646868 10.4161/cc.10.12.16002 PMC3154420

[5] Franco OE, Jiang M, Strand DW, Peacock J, Fernandez S, et al (2011) Altered TGF-β signaling in a subpopulation of human stromal cells promotes prostatic carcinogenesis. Cancer Res 71: 1272–81. 21303979 10.1158/0008-5472.CAN-10-3142 PMC3076790

[6] Wallace JA, Li F, Leone G, Ostrowski MC (2011) Pten in the breast tumor microenvironment: modeling tumor-stroma coevolution. Cancer Res 71: 1203–7. 21303970 10.1158/0008-5472.CAN-10-3263 PMC3075554

[7] Salem AF, Whitaker-Menezes D, Lin Z, Martinez-Outschoorn UE, Tanowitz HB, et al (2012) Two-compartment tumor metabolism: autophagy in the tumor microenvironment and oxidative mitochondrial metabolism (OXPHOS) in cancer cells. Cell Cycle 11: 2545–56. 22722266 10.4161/cc.20920 PMC3404881

[8] Taipale J, Beachy PA (2001) The Hedgehog and Wnt signalling pathways in cancer. Nature 411: 349–54. 11357142 10.1038/35077219

[9] Varjosalo M, Taipale J (2008) Hedgehog: functions and mechanisms. Genes Dev 22: 2454–72. 18794343 10.1101/gad.1693608

[10] Stecca B, Ruiz I Altaba A (2010) Context-dependent regulation of the GLI code in cancer by HEDGEHOG and non-HEDGEHOG signals. J Mol Cell Biol 2: 84–95. 20083481 10.1093/jmcb/mjp052 PMC2905064

[11] Beachy PA, Hymowitz SG, Lazarus RA, Leahy DJ, Siebold C (2010) Interactions between Hedgehog proteins and their binding partners come into view. Genes Dev 24: 2001–12. 20844013 10.1101/gad.1951710 PMC2939362

[12] Wang C, Pan Y, Wang B (2010) Suppressor of fused and Spop regulate the stability, processing and function of Gli2 and Gli3 full-length activators but not their repressors. Development 137: 2001–9. 20463034 10.1242/dev.052126 PMC2875842

[13] Chinchilla P, Xiao L, Kazanietz MG, Riobo NA (2010) Hedgehog proteins activate pro-angiogenic responses in endothelial cells through non-canonical signaling pathways. Cell Cycle 9: 570–9. 20081366 10.4161/cc.9.3.10591

[14] Nolan-Stevaux O, Lau J, Truitt ML, Chu GC, Hebrok M, et al (2009) GLI1 is regulated through Smoothened-independent mechanisms in neoplastic pancreatic ducts and mediates PDAC cell survival and transformation. Genes Dev 23: 24–36. 19136624 10.1101/gad.1753809 PMC2632164

[15] Bolaños AL, Milla CM, Lira JC, Ramírez R, Checa M, et al (2012) Role of Sonic Hedgehog in idiopathic pulmonary fibrosis. Am J Physiol Lung Cell Mol Physiol 303: L978–990. 23023967 10.1152/ajplung.00184.2012

[16] Cigna N, Farrokhi Moshai E, Brayer S, Marchal-Somme J, Wémeau-Stervinou L, et al (2012) The hedgehog system machinery controls transforming growth factor-β-dependent myofibroblastic differentiation in humans: involvement in idiopathic pulmonary fibrosis. Am J Pathol 181: 2126–37. 23031257 10.1016/j.ajpath.2012.08.019

[17] Li Y, Li Y, Yang T, Wei S, Wang J, et al (2013) Clinical Significance of EML4-ALK Fusion Gene and Association with EGFR and KRAS Gene Mutations in 208 Chinese Patients with Non-Small Cell Lung Cancer. PLoS ONE 8: e52093. 23341890 10.1371/journal.pone.0052093 PMC3544857

[18] Pu X, Ye Y, Spitz MR, Wang L, Gu J, et al (2012) Predictors of survival in never-smokers with non-small cell lung cancer: a large-scale, two-phase genetic study. Clin Cancer Res 18: 5983–91. 22977190 10.1158/1078-0432.CCR-12-0774 PMC3640870

[19] Li G-H, Cui Y-S, Wu Q-Y, Zhang X-J, Gao Y-F (2013) Clinicopathologic significance of β-catenin and matrix metalloproteinase-2 expression in non-small cell lung cancer. Med Oncol 30: 437. 23292837 10.1007/s12032-012-0437-z

[20] Watkins DN, Berman DM, Burkholder SG, Wang B, Beachy PA, et al (2003) Hedgehog signalling within airway epithelial progenitors and in small-cell lung cancer. Nature 422: 313–7. 12629553 10.1038/nature01493

[21] Park K-S, Martelotto LG, Peifer M, Sos ML, Karnezis AN, et al (2011) A crucial requirement for Hedgehog signaling in small cell lung cancer. Nat Med 17: 1504–8. 21983857 10.1038/nm.2473 PMC3380617

[22] Low W-C, Wang C, Pan Y, Huang X-Y, Chen JK, et al (2008) The decoupling of Smoothened from Galphai proteins has little effect on Gli3 protein processing and Hedgehog-regulated chick neural tube patterning. Dev Biol 321: 188–96. 18590719 10.1016/j.ydbio.2008.06.014 PMC2597282

[23] Yuan Z, Goetz JA, Singh S, Ogden SK, Petty WJ, et al (2007) Frequent requirement of hedgehog signaling in non-small cell lung carcinoma. Oncogene 26: 1046–55. 16909105 10.1038/sj.onc.1209860

[24] Zhang X, Harrington N, Moraes RC, Wu M-F, Hilsenbeck SG, et al (2009) Cyclopamine inhibition of human breast cancer cell growth independent of Smoothened (Smo). Breast Cancer Res Treat 115: 505–21. 18563554 10.1007/s10549-008-0093-3 PMC5300001

[25] Meyers-Needham M, Lewis JA, Gencer S, Sentelle RD, Saddoughi SA, et al (2012) Off-target function of the Sonic hedgehog inhibitor cyclopamine in mediating apoptosis via nitric oxide-dependent neutral sphingomyelinase 2/ceramide induction. Mol Cancer Ther 11: 1092–102. 22452947 10.1158/1535-7163.MCT-11-0705 PMC3709261

[26] Slusarz A, Shenouda NS, Sakla MS, Drenkhahn SK, Narula AS, et al (2010) Common botanical compounds inhibit the hedgehog signaling pathway in prostate cancer. Cancer Res 70: 3382–90. 20395211 10.1158/0008-5472.CAN-09-3012

[27] Bai CB, Joyner AL (2001) Gli1 can rescue the in vivo function of Gli2. Development 128: 5161–72. 11748151 10.1242/dev.128.24.5161

[28] Dennler S, André J, Alexaki I, Li A, Magnaldo T, et al (2007) Induction of sonic hedgehog mediators by transforming growth factor-beta: Smad3-dependent activation of Gli2 and Gli1 expression in vitro and in vivo. Cancer Res 67: 6981–6. 17638910 10.1158/0008-5472.CAN-07-0491

[29] Dennler S, André J, Verrecchia F, Mauviel A (2009) Cloning of the human GLI2 Promoter: transcriptional activation by transforming growth factor-beta via SMAD3/beta-catenin cooperation. J Biol Chem 284: 31523–31. 19797115 10.1074/jbc.M109.059964 PMC2797221

[30] Johnson RW, Nguyen MP, Padalecki SS, Grubbs BG, Merkel AR, et al (2011) TGF-beta promotion of Gli2-induced expression of parathyroid hormone-related protein, an important osteolytic factor in bone metastasis, is independent of canonical Hedgehog signaling. Cancer Res 71: 822–31. 21189326 10.1158/0008-5472.CAN-10-2993 PMC3077118

[31] Fan Q, He M, Sheng T, Zhang X, Sinha M, et al (2010) Requirement of TGFbeta signaling for SMO-mediated carcinogenesis. J Biol Chem 285: 36570–6. 20858897 10.1074/jbc.C110.164442 PMC2978585

[32] Pan Y, Bai CB, Joyner AL, Wang B (2006) Sonic hedgehog signaling regulates Gli2 transcriptional activity by suppressing its processing and degradation. Mol Cell Biol 26: 3365–77. 16611981 10.1128/MCB.26.9.3365-3377.2006 PMC1447407

[33] Yoon JW, Kita Y, Frank DJ, Majewski RR, Konicek BA, et al (2002) Gene expression profiling leads to identification of GLI1-binding elements in target genes and a role for multiple downstream pathways in GLI1-induced cell transformation. J Biol Chem 277: 5548–55. 11719506 10.1074/jbc.M105708200

[34] Chi S, Huang S, Li C, Zhang X, He N, et al (2006) Activation of the hedgehog pathway in a subset of lung cancers. Cancer Lett 244: 53–60. 16446029 10.1016/j.canlet.2005.11.036

[35] Motoyama J, Liu J, Mo R, Ding Q, Post M, et al (1998) Essential function of Gli2 and Gli3 in the formation of lung, trachea and oesophagus. Nat Genet 20: 54–7. 9731531 10.1038/1711

[36] Brentnall TA, Lai LA, Coleman J, Bronner MP, Pan S, et al (2012) Arousal of cancer-associated stroma: overexpression of palladin activates fibroblasts to promote tumor invasion. PLoS ONE 7: e30219. 22291919 10.1371/journal.pone.0030219 PMC3264580

[37] Coussens LM, Tinkle CL, Hanahan D, Werb Z (2000) MMP-9 supplied by bone marrow-derived cells contributes to skin carcinogenesis. Cell 103: 481–90. 11081634 10.1016/s0092-8674(00)00139-2 PMC2843102

[38] Giraudo E, Inoue M, Hanahan D (2004) An amino-bisphosphonate targets MMP-9-expressing macrophages and angiogenesis to impair cervical carcinogenesis. J Clin Invest 114: 623–33. 15343380 10.1172/JCI22087 PMC514591

[39] Van Kempen LCL, Coussens LM (2002) MMP9 potentiates pulmonary metastasis formation. Cancer Cell 2: 251–2. 12398887 10.1016/s1535-6108(02)00157-5

[40] Yoo YA, Kang MH, Lee HJ, Kim B, Park JK, et al (2011) Sonic hedgehog pathway promotes metastasis and lymphangiogenesis via activation of Akt, EMT, and MMP-9 pathway in gastric cancer. Cancer Res 71: 7061–70. 21975935 10.1158/0008-5472.CAN-11-1338

[41] Levental KR, Yu H, Kass L, Lakins JN, Egeblad M, et al (2009) Matrix crosslinking forces tumor progression by enhancing integrin signaling. Cell 139: 891–906. 19931152 10.1016/j.cell.2009.10.027 PMC2788004

[42] Moreno-Barriuso N, López-Malpartida AV, De Pablo F, Pichel JG (2006) Alterations in alveolar epithelium differentiation and vasculogenesis in lungs of LIF/IGF-I double deficient embryos. Dev Dyn 235: 2040–50. 16691571 10.1002/dvdy.20842

[43] Loewen GM, Tracy E, Blanchard F, Tan D, Yu J, et al (2005) Transformation of human bronchial epithelial cells alters responsiveness to inflammatory cytokines. BMC Cancer 5: 145. 16271139 10.1186/1471-2407-5-145 PMC1289280

[44] Wysoczynski M, Miekus K, Jankowski K, Wanzeck J, Bertolone S, et al (2007) Leukemia inhibitory factor: a newly identified metastatic factor in rhabdomyosarcomas. Cancer Res 67: 2131–40. 17332343 10.1158/0008-5472.CAN-06-1021

[45] Chen W, Tang T, Eastham-Anderson J, Dunlap D, Alicke B, et al (2011) Canonical hedgehog signaling augments tumor angiogenesis by induction of VEGF-A in stromal perivascular cells. Proc Natl Acad Sci U S A 108: 9589–94. 21597001 10.1073/pnas.1017945108 PMC3111273

[46] Moran CM, Myers CT, Lewis CM, Krieg PA (2012) Hedgehog regulates angiogenesis of intersegmental vessels through the VEGF signaling pathway. Dev Dyn 241: 1034–42. 22513894 10.1002/dvdy.23795 PMC4747427

[47] Yamazaki M, Nakamura K, Mizukami Y, Ii M, Sasajima J, et al (2008) Sonic hedgehog derived from human pancreatic cancer cells augments angiogenic function of endothelial progenitor cells. Cancer Sci 99: 1131–8. 18422746 10.1111/j.1349-7006.2008.00795.x PMC11158306

[48] Seton-Rogers S (2011) Cancer stem cells. VEGF promotes stemness. Nat Rev Cancer 11: 831. 10.1038/nrc3176 22089417

[49] Shin JE, Park SH, Jang YK (2011) Epigenetic up-regulation of leukemia inhibitory factor (LIF) gene during the progression to breast cancer. Mol Cells 31: 181–9. 21191816 10.1007/s10059-011-0020-z PMC3932684

[50] O’Toole SA, Machalek DA, Shearer RF, Millar EKA, Nair R, et al (2011) Hedgehog overexpression is associated with stromal interactions and predicts for poor outcome in breast cancer. Cancer Res 71: 4002–14. 21632555 10.1158/0008-5472.CAN-10-3738

[51] Stewart GA, Hoyne GF, Ahmad SA, Jarman E, Wallace WAH, et al (2003) Expression of the developmental Sonic hedgehog (Shh) signalling pathway is up-regulated in chronic lung fibrosis and the Shh receptor patched 1 is present in circulating T lymphocytes. J Pathol 199: 488–95. 12635140 10.1002/path.1295

[52] Kinzel D, Boldt K, Davis EE, Burtscher I, Trümbach D, et al (2010) Pitchfork regulates primary cilia disassembly and left-right asymmetry. Dev Cell 19: 66–77. 20643351 10.1016/j.devcel.2010.06.005 PMC3671612

[53] Hruz T, Laule O, Szabo G, Wessendorp F, Bleuler S, et al (2008) Genevestigator v3: a reference expression database for the meta-analysis of transcriptomes. Adv Bioinformatics 2008: 420747. 19956698 10.1155/2008/420747 PMC2777001

