# Supplementary figures and images for: Gli1 Mediates Lung Cancer Cell Proliferation and Sonic Hedgehog-Dependent Mesenchymal Cell Activation
Source: PLoS One. 2013 May 7;8(5):e63226. doi: 10.1371/journal.pone.0063226 (PMC3646741; doi:10.1371/journal.pone.0063226)

Supplementary Figure 1

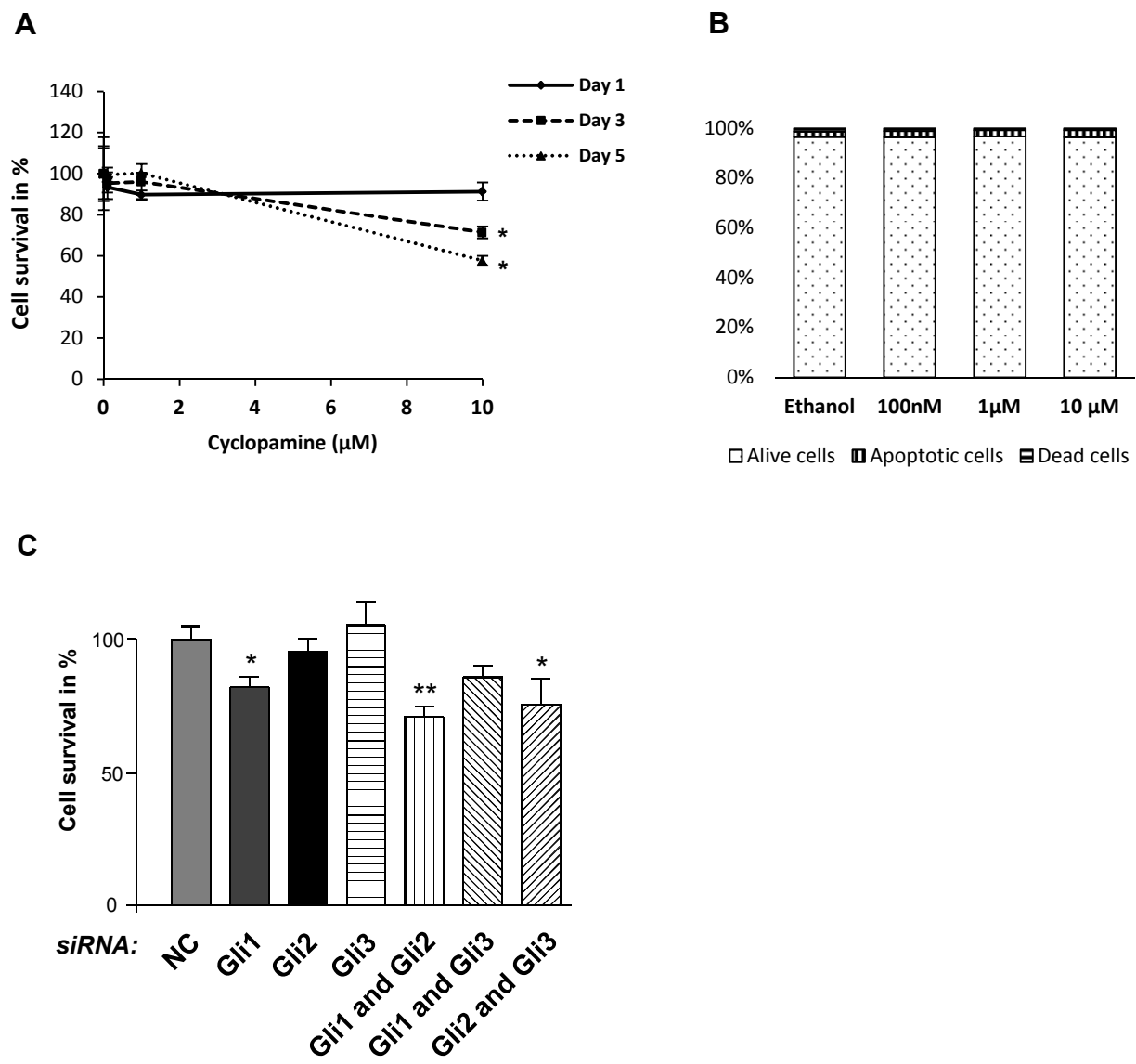

Supplement: Figure S1 — Inhibition of Hedgehog signaling decreases A549 cell survival. (A) Lung adenocarcinoma A549 cells were cultured in absence or presence of 100 nM, 1 µM or 10 µM of cyclopamine for 1, 3 and 5 days. Cell survival was assessed by MTT assay and is expressed in percentage relative to non-treated cells. *p<0,1. (B) The proportion of A549 apoptotic and dead cells upon 72 hours of cyclopamine treatment (100 nM, 1 µM or 10 mM) was assessed using annexin V/PI staining and flow cytometry. The percentage of alive cells, apoptotic and dead cells, from the gated population are presented. (C) A549 Cells were transfected with one or two siRNA at the same time, as indicated. Cell survival was assessed by MTT assay 72 hours after the transfection. Results are presented as relative cell survival compared with cells transfected with the negative control siRNA (NC). *p<0,1; **p<0,05. (PDF) [file pone.0063226.s001.pdf]

## Supplementary Figure 2

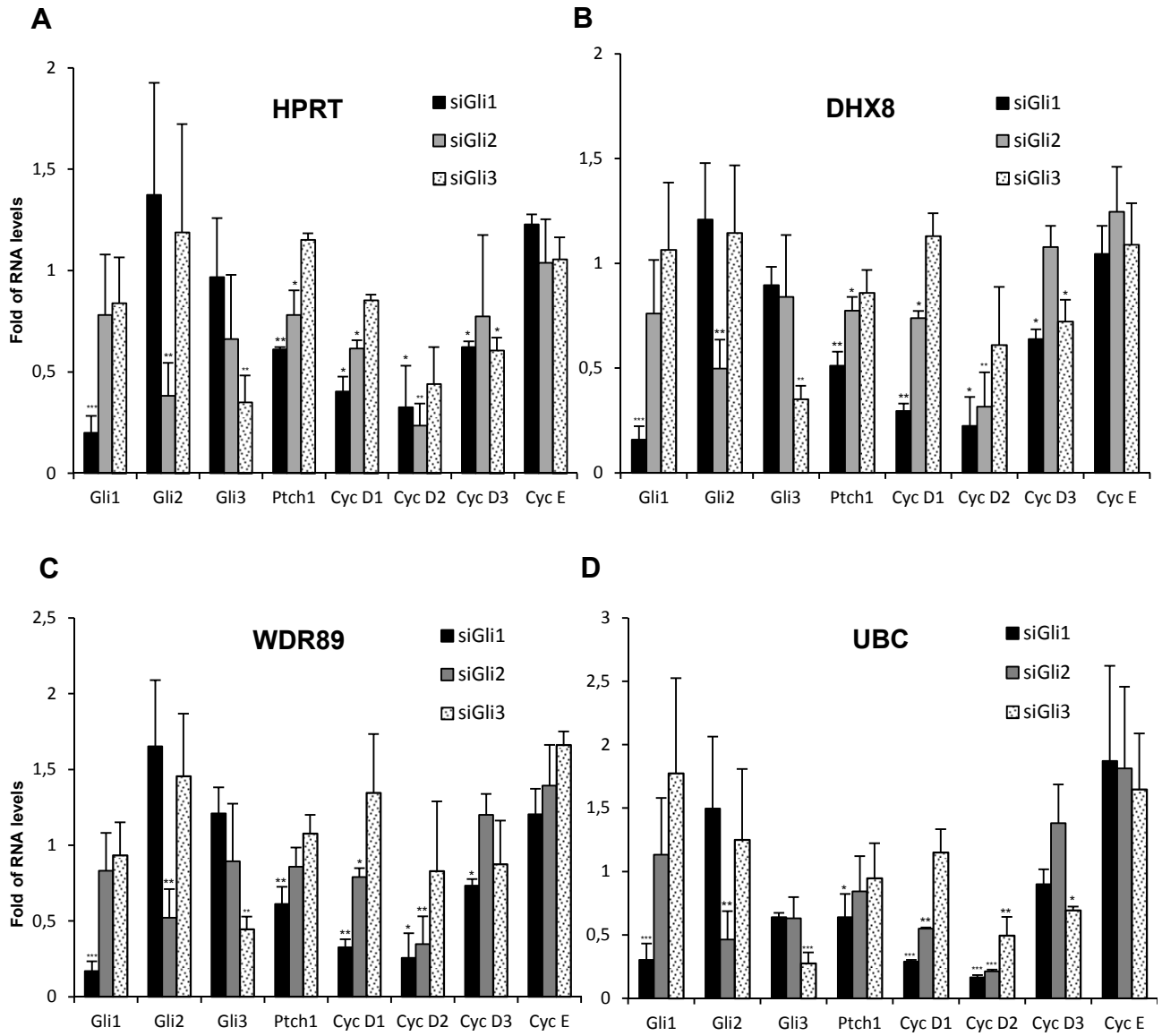

Supplement: Figure S2 — The pattern of expression of Shh-related genes and cyclins upon Gli knockdown in A549 cells is similar when different reference genes are used. The knockdown of Gli1, Gli2 or Gli3 was performed in A549 cells using siRNA. The specific silencing of each human transcription factor Gli and the effect of the silencing of each Gli in the expression of Hedgehog receptor Ptch1 and in the G1/S phase cyclins D (Cyc D1, Cyc D2, Cyc D3) and cyclin E (Cyc E1) was studied by RT-qPCR. Relative transcript abundance of a gene (vertical axes) is expressed as fold of relative changes in mRNA levels (2∧∧Ct) compared with cells transfected with a negative control siRNA (NC siRNA) having no homology in vertebrate transcriptome. Relative mRNA levels were calculated taking four different genes for reference: Hprt1 (A), Dhx8 (B), Wdr89(C) or Ubc (D). *p<0,1; **p<0,05; ***p<0,01. (PDF) [file pone.0063226.s002.pdf]

## Supplementary Figure 3

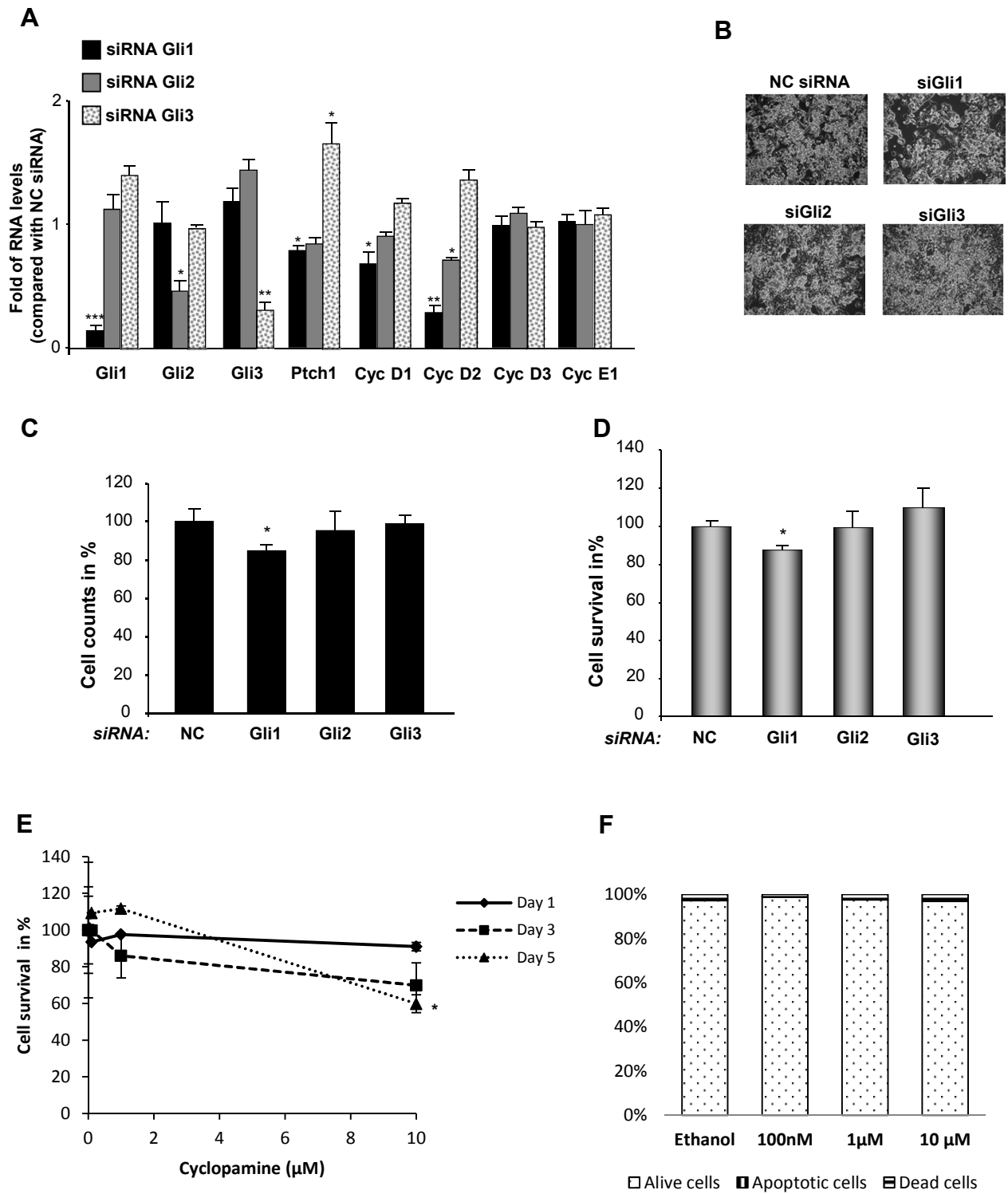

Supplement: Figure S3 — Silencing of Gli1 decreases lung cancer squamous H520 cell proliferation, cyclin D1 and cyclin D2 expression. The knockdown of Gli1, Gli2 or Gli3 was performed in H520 cells using siRNA. (A) The specific silencing of each human transcription factor Gli and the effect of the silencing of each Gli in the expression of Hedgehog receptor Ptch1 and in the G1/S phase cyclins D (Cyc D1, Cyc D2, Cyc D3) and cyclin E (Cyc E1) was studied by RT-qPCR. *p<0,1; **p<0,05; ***p<0,01. (B) Representative phase-contrast microscopic pictures after 72hours of siRNA are presented. The impact of silencing Gli1, Gli2 or Gli3 in H520 cell proliferation was assessed by cell counting (C) and in cell survival by MTT assay (D). Results are presented in percentage as relative proliferation and relative survival compared with cells transfected with the negative control siRNA (NC). *p<0,1. (E) H520 cells were cultured in absence or presence of 100 nM, 1 µM or 10 µM of cyclopamine for 1, 3 and 5 days. Cell survival was assessed by MTT assay and is expressed in percentage relative to non-treated cells. *p<0, 1 (F) The proportion of H520 apoptotic and dead cells upon 72 hours of cyclopamine treatment (100 nM, 1 µM or 10 mM) was assessed by using annexin V/PI staining and flow cytometry. The percentage of alive, apoptotic and dead cells, from the gated population are presented. (PDF) [file pone.0063226.s003.pdf]

## Supplementary Figure 4

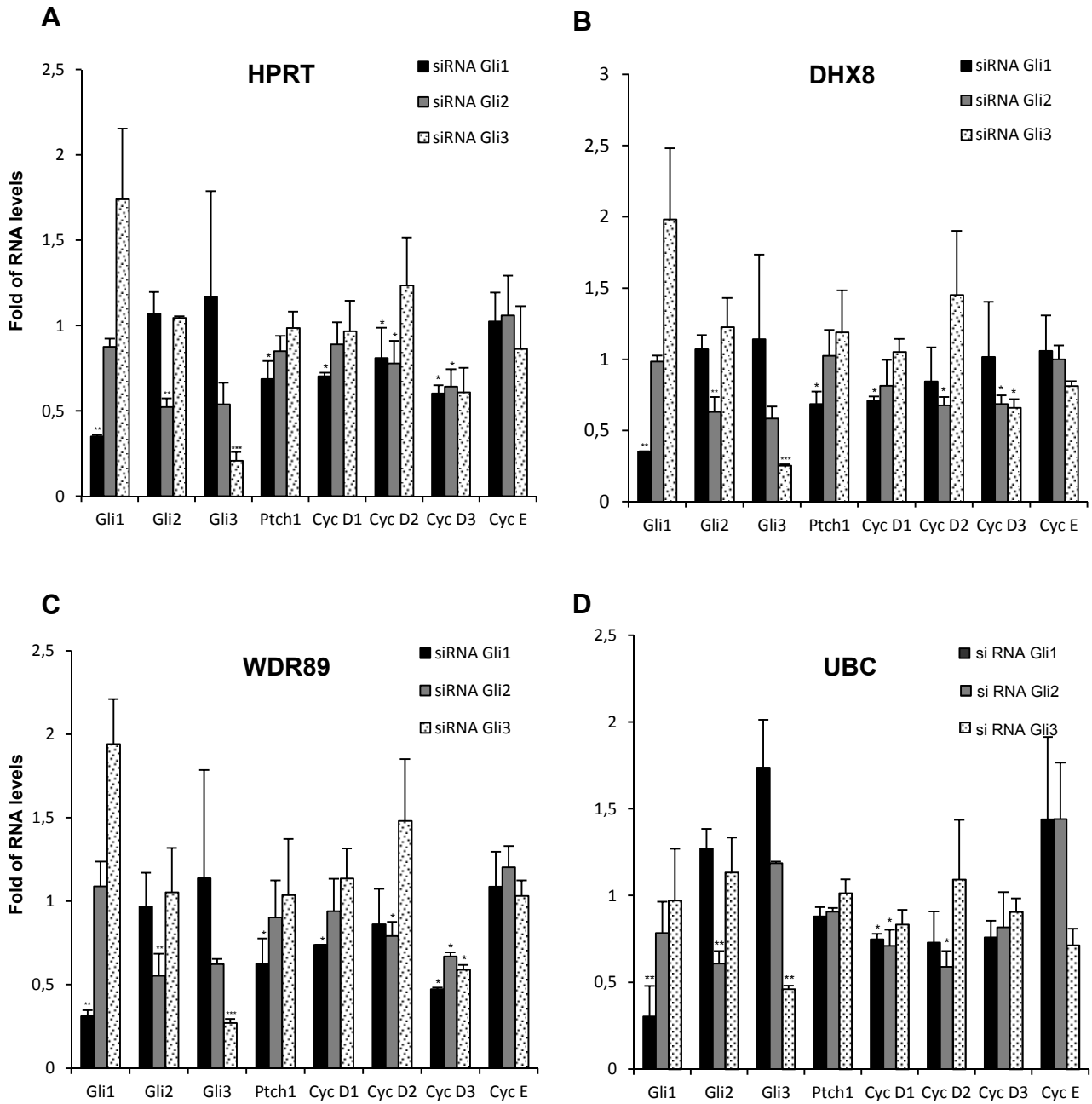

Supplement: Figure S4 — The pattern of expression of Shh-related genes and cyclins upon Gli knockdown in H520 cells is similar when different reference genes are used. The knockdown of Gli1, Gli2 or Gli3 was performed in H520 cells using siRNA. The specific silencing of each human transcription factor Gli and the effect of the silencing of each Gli in the expression of Hedgehog receptor Ptch1 and in the G1/S phase cyclins D (Cyc D1, Cyc D2, Cyc D3) and cyclin E (Cyc E1) was studied by RT-qPCR. Relative transcript abundance of a gene (vertical axes) is expressed as fold of relative changes in mRNA levels (2∧∧Ct) compared with cells transfected with a negative control siRNA (NC siRNA) having no homology in vertebrate transcriptome. Relative mRNA levels were calculated taking four different genes for reference: Hprt1 (A), Dhx8 (B), Wdr89(C) or Ubc (D). *p<0,1; **p<0,05; ***p<0,01. (PDF) [file pone.0063226.s004.pdf]

**Supplementary Figure 5**

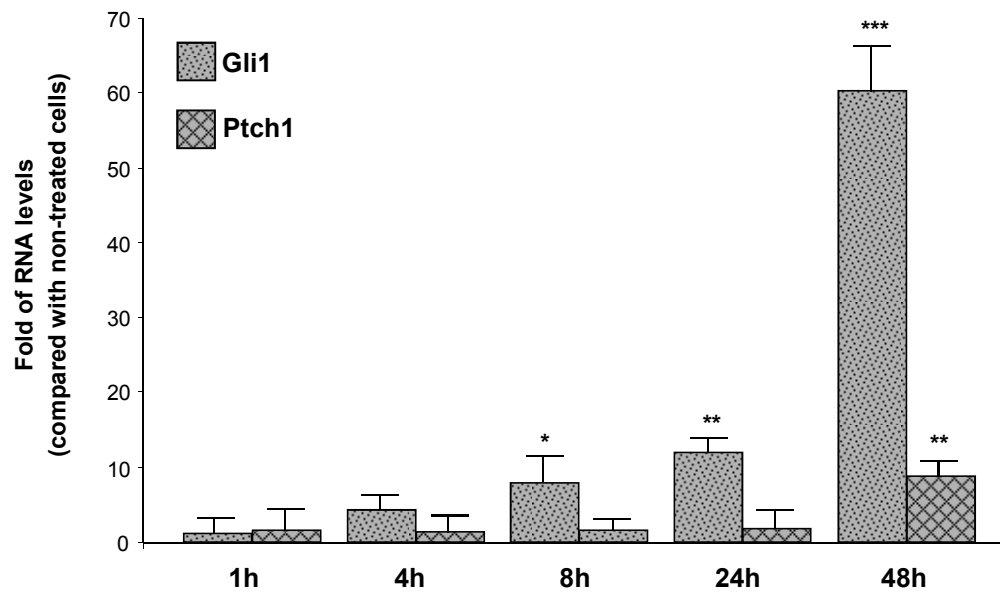

Supplement: Figure S5 — Mouse primary limb buds cells were used as a positive control for exogenous Shh treatment. Primary limb buds cells from mouse embryo were serum-starved for 24 hours and then treated or not with mouse Shh (500 ng/ml) for the indicated times. Gli1 and Ptch1 mRNA levels were evaluated by RT-qPCR. Relative transcript abundance of a gene is expressed as fold of relative changes in mRNA levels (2∧∧Ct) compared with non-treated cells for each time point. *p<0,1; **p<0,05; ***p<0,01. *p<0,1;**p<0,05; ***p<0,01. (PDF) [file pone.0063226.s005.pdf]

**Supplementary Figure 6**

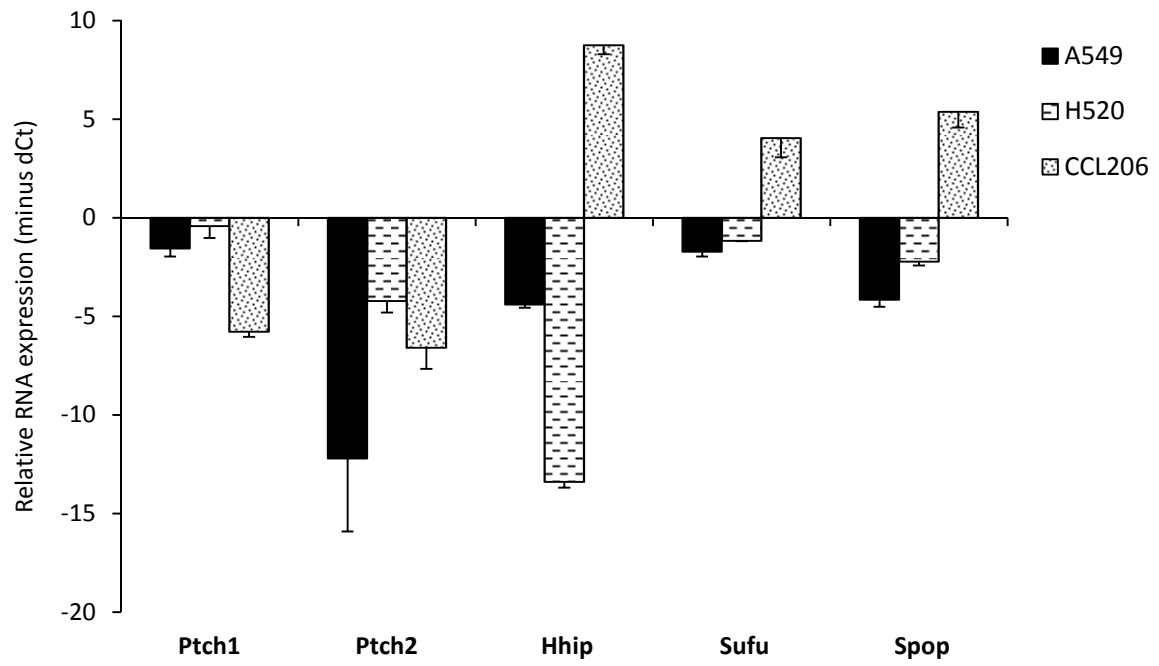

Supplement: Figure S6 — The relative expression of Ptch, Hhip, Sufu and Spop in NSCLC cells and CCL206 lung fibroblasts. The relative mRNA expression of Ptch1, Ptch2, Hhip, Sufu and Spop was assessed by RT-qPCR in cells cultured in medium containing 1% of Fcs. Relative transcript abundance of a gene is expressed as minus dCt (dCt = Ct of gene of interest – Ct reference gene) compared with Hprt1. (PDF) [file pone.0063226.s006.pdf]

## Supplementary Figure 7

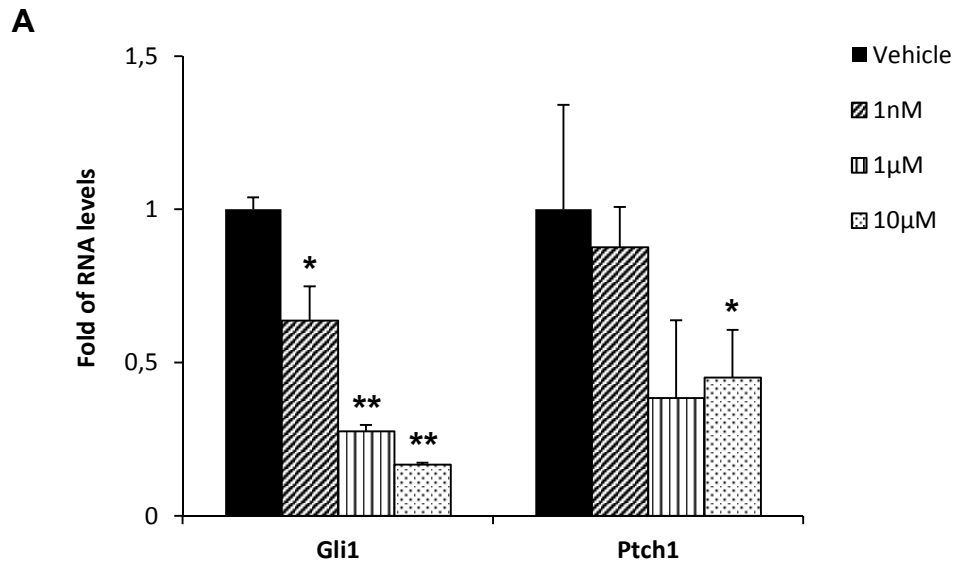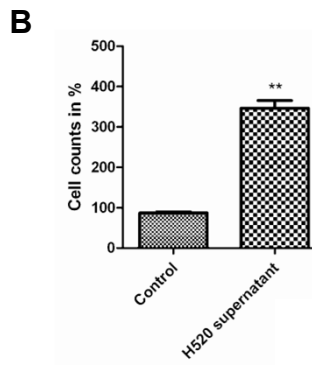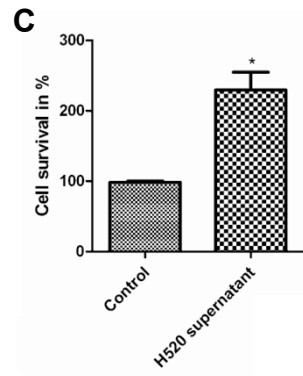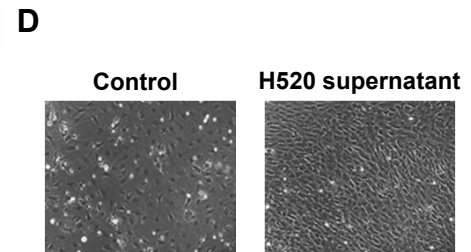

Supplement: Figure S7 — Shh pathway correlates with lung fibroblast proliferation and cell survival. (A) Cyclopamine reduces Gli1 and Ptch1 expression in CCL206 lung fibroblasts. CCL206 fibroblasts were treated or not with 1 nM, 1 µM or 10 µM of cyclopamine for 72 h. Gli1 and Ptch1 mRNA levels were evaluated by RT-qPCR. Relative transcript abundance of each gene is expressed as fold of relative changes in mRNA levels compared to non-treated cells. *p<0,1; **p<0,05. Lung fibroblasts were cultured for 5 days with normal medium or with the supernatant of H520 cells containing 0,5% of Fcs. Cell proliferation was assessed by cell counting (B) and cell survival by MTT assay (C). Results are presented in percentage as relative proliferation and relative survival compared with control condition (fibroblasts grown in normal medium). *p<0,1; **p<0,05. (D) Representative phase-contrast microscope pictures of fibroblasts upon treatment are shown. (PDF) [file pone.0063226.s007.pdf]

Supplementary Figure 8

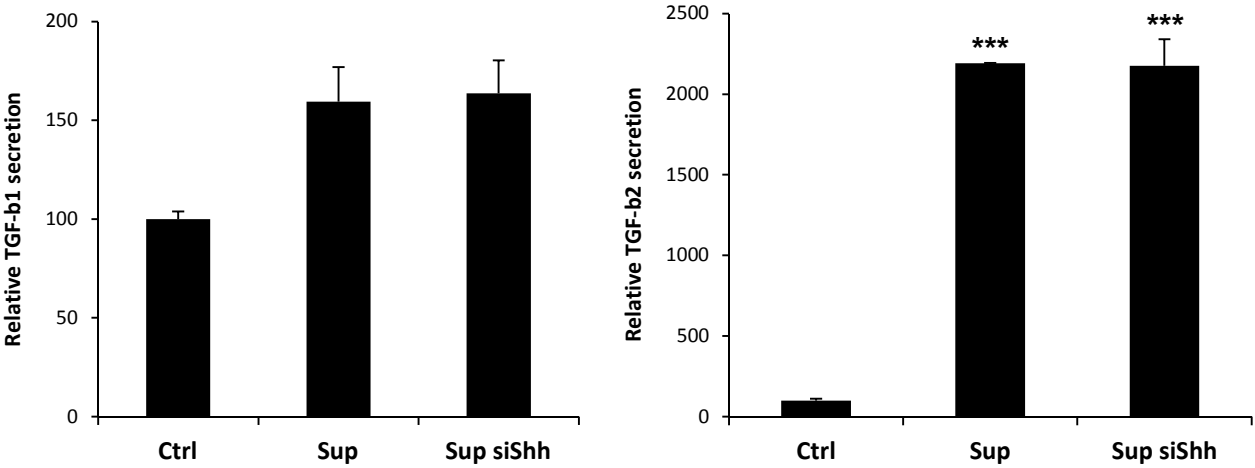

Supplement: Figure S8 — TGF-ß secretion in lung fibroblasts is increased by NSCLC supernatant but does not depend on Shh. CCL206 lung fibroblasts were cultured or not with the supernatant of H520 transfected with a NC siRNA(Sup) or with Shh siRNA (Sup siShh) for 48 h. Levels of secreted TGF-b1 (A) and TGF-b2 (B) were evaluated in CCL206 supernatant by multiplex biometric ELISA-based immunoassay (Bioplex system). Results are presented in percentage as relative secretion compared with cells cultured without H520 supernatant. ***p<0,01. (PDF) [file pone.0063226.s008.pdf]
