# Supplementary material for: Gli1 Mediates Lung Cancer Cell Proliferation and Sonic Hedgehog-Dependent Mesenchymal Cell Activation
Source: PLoS One. 2013 May 7;8(5):e63226. doi: 10.1371/journal.pone.0063226 (PMC3646741; doi:10.1371/journal.pone.0063226)
Supplement: Table S1 — Primer sequences. (DOCX) [file pone.0063226.s009.docx]

**Supporting Table S1.** Primer sequences.

| **Gene** | **Accession** | **Specie** | **Forward primer (5´>3´)** | **Reverse primer (5´>3´)** |
| --- | --- | --- | --- | --- |
| Hprt1 | NM_000194.2 | Human | AAGGACCCCACGAAGTGTTG | GGCTTTGTATTTTGCTTTTCCA |
| Gli1 | NM_001160045.1 | Human | TTCCTACCAGAGTCCCAAGT | CCCTATGTGAAGCCCTATTT |
| Gli2 | NM_005270.4 | Human | ATCTGGACAGGGATGACTGTAAGC | TGTCGTACTCCTTGGTGCAGTCTT |
| Gli3 | NM_000168.5 | Human | TGCAGGGTGAATGGTATCAA | TGATTAGCACCTGGGGAAAG |
| Shh | NM_000193.2 | Human | ACCGAGGGCTGGGACGAAGA | ATTTGGCCGCCACCGAGTT |
| Ihh | NM_002181.3 | Human | ACTTCTGCCTGGTCCTGTTG | AGCGATCTTGCCTTCATAGC |
| Dhh | NM_021044.2 | Human | TGATGACCGAGCGTTGTAAG | GCCAGCAACCCATACTTGTT |
| Ptch1 | NM_008957.2 | Human | GCGGGATCTGAGTTCGACTTCATT | GGAAGCAAAACCAGCCCATTGAGA |
| Ptch2 | NM_003738.4 | Human | AAGTCCAAGTATCACTCTATG | TCATCCGCTCAATCATTC |
| Sufu | NM_016169.3 | Human | CGGAGGGGAGAGACCATATT | CACTTGGCACTGACACCACT |
| Spop | NM_001007226.1 | Human | TGTTTCACTCACCTGCCTTGTCTC | ACAAATCCCTGCGACTCATTAGGC |
| Hhip | NM_022475.2 | Human | TCTCAAAGCCTGTTCCACTCA | AAGCACAACCCACCATCTTTT |
| Cyclin D1 | NM_053056.2 | Human | GGTGAACAAGCTCAAGTGGAACCT | GCGTGTTTGCGGATGATCTGTTTG |
| Cyclin D2 | NM_001759.3 | Human | GTTCCTGGCCTCCAAACTCAAAGA | GGCTTGATGGAGTTGTCGGTGTAA |
| Cyclin D3 | NM_001136017.2 | Human | TGCTGGTCCTAGGGAAGCTCAA | GTGCAGAATGAAGGCCAGGAAATC |
| Cyclin E1 | NM_001238.2 | Human | GTTGCACCAGTTTGCGTATGTGAC | AAACGCCACTTAAGGGCCTTCA |
| WDR89 | NM_001008726.2 | Human | AGTACGTTTCCATCCCAGCAATCC | AGGCCATCAGATGAACCTGAGACT |
| DHX8 | NM_004941.1 | Human | TGACCCAGAGAAGTGGGAGA | ATCTCAAGGTCCTCATCTTCTTCA |
| UBC | NM_021009.5 | Human | CACTTGGTCCTGCGCTTGA | TTTTTTGGGAATGCAACAACTT |
| Hprt1 | NM_013556 | Mouse | cct aag atg agc gca agt tga a | ccacaggactagaacacctgctaa |
| Gli1 | NM_010296.2 | Mouse | CTGGAGAACCTTAGGCTGGA | CGGCTGACTGTGTAAGCAGA |
| Gli2 | NM_001081125.1 | Mouse | ctgtgcagtggaatgaggtgagtt | tatcctggatactggccaaaggct |
| Gli3 | NM_008130.2 | Mouse | gcaagtggttcctatgggcactta | ttgctgtcggcttaggatctgttg |
| Ptch1 | NM_008957.2 | Mouse | AATCTGGGTCGGCTGGGTTGAGGC | ATAACAGGCAATGGAAGTTGGAAC |
| Ptch2 | NM_008958.2 | Mouse | ACCTAGTGCTCCCAACCGGC | AGATCTCTGGCTGTGCCCCCT |
| Sufu | NM_015752.2 | Mouse | CATCAGCTTTGGCCTGAGTGATCT | CCAAATCCACTTGGTCCGTCTGTT |
| Spop | NM_025287.2 | Mouse | CTTTGGCCTGACCAAGTTTGACAC | TTTTGCTCCAGGCACTGACGAT |
| Hhip | NM_020259.4 | Mouse | TTCAGTAACGGCCCTTTGGTTGGT | CGGCTTTTCTTGCCATTGCTTG |
| α-SMA | NM_007392.2 | Mouse | GCTGGTGATGATGCTCCCA | GCCCATTCCAACCATTACTCC |
| Col4a1 | NM_009931.2 | Mouse | GGCTCTCCGGGTTCAATAGG | CACCAACAGGACCAATGGG |
| MMP2 | NM_008610.2 | Mouse | TCTGGTGCTCCACCACATACAACT | CTGCATTGCCACCCATGGTAAACA |
| MMP9 | NM_013599.2 | Mouse | TGAGCTGGACAGCCAGACACTAAA | TCGCGGCAAGTCTTCAGAGTAGTT |
